# The NONRATT023402.2/rno-miR-3065-5p/NGFR axis affects levodopa-induced dyskinesia in a rat model of Parkinson’s disease

**DOI:** 10.1038/s41420-023-01644-2

**Published:** 2023-09-15

**Authors:** Qiao Wang, Huizhi Wang, Wenjia Meng, Chong Liu, Renpeng Li, Moxuan Zhang, Kun Liang, Yuan Gao, Tingting Du, Jianguo Zhang, Chunlei Han, Lin Shi, Fangang Meng

**Affiliations:** 1 Department of Functional Neurosurgery, Beijing Neurosurgical Institute, Capital Medical University, Beijing, China; 2 Beijing Key Laboratory of Neurostimulation, Beijing, China; 3 Beijing Hospital, National Center of Gerontology, Institute of Geriatric Medicine, Chinese Academy of Medical Sciences, Beijing, China; 4 Clinical School, Tianjin Medical University, Tianjin, China; 5 Department of Neurosurgery, Beijing Tiantan Hospital, Capital Medical University, Beijing, China; 6 Chinese Institute for Brain Research, Beijing, China

**Keywords:** Epigenetics and behaviour, Parkinson's disease

## Abstract

Levodopa-induced dyskinesia (LID) is a common motor complication in Parkinson’s disease. However, few studies have focused on the pathogenesis of LID at the transcriptional level. NONRATT023402.2, a long non-coding RNA (lncRNA) that may be related to LID was discovered in our previous study and characterized in rat models of LID. In the present study, NONRATT023402.2 was overexpressed by injection of adeno-associated virus (AAV) in striatum of LID rats, and 48 potential target genes, including nerve growth factor receptor (*NGFR*) were screened using next-generation sequencing and target gene predictions. The NONRATT023402.2/rno-miR-3065-5p/*NGFR* axis was verified using a dual luciferase reporter gene. Overexpression of NONRATT023402.2 significantly increased the abnormal involuntary movements (AIM) score of LID rats, activated the PI3K/Akt signaling pathway, and up-regulated c-Fos in the striatum. *NGFR* knockdown by injection of ShNGFR-AAV into the striatum of LID rats resulted in a significant decrease in the PI3K/Akt signaling pathway and c-Fos expression. The AIM score of LID rats was positively correlated with the expressions of NONRATT023402.2 and *NGFR*. A dual luciferase reporter assay showed that c-Fos, as a transcription factor, bound to the NONRATT023402.2 promoter and activated its expression. Together, the results showed that NONRATT023402.2 regulated *NGFR* expression via a competing endogenous RNA mechanism, which then activated the PI3K/Akt pathway and promoted c-Fos expression. This suggested that c-Fos acted as a transcription factor to activate NONRATT023402.2 expression, and form a positive feedback regulation loop in LID rats, thus, aggravating LID symptoms. NONRATT023402.2 is therefore a possible novel therapeutic target for LID.

## Introduction

Parkinson’s disease (PD) is a common neurodegenerative disease in older adults that affects 1 − 2/1000 people [1]. The main treatment of PD is exogenous supplementation of levodopa (L-DOPA), but long-term L-DOPA treatment leads to levodopa-induced dyskinesia (LID). LID is characterized by abnormal involuntary movements (AIM), which seriously affect the quality of life of PD patients. Long-term pulsed L-DOPA stimulation can lead to LID, causing presynaptic nigrostriatal system degeneration with a relatively preserved postsynaptic nigrostriatal system [2]. An inherent difficulty in the treatment of PD involves how to maximize the control of PD motor symptoms while avoiding LID. At present, the lack of understanding of the specific mechanism of LID still hinders development of an effective treatment for PD.

Long non-coding RNAs (lncRNAs) are a type of RNA more than 200 nucleotides in length, with no protein coding capacity, but with the ability to regulate gene expression at the transcriptional, posttranscriptional, and epigenetic levels [3, 4]. According to the competing endogenous RNAs (ceRNAs)-mediated regulation mechanism, lncRNAs in the cytoplasm may function as miRNA sponges to abrogate the inhibitory impact of miRNAs on target genes [5, 6]. Noncoding RNA crosstalk is therefore thought to play a major role in the development and progression of central nervous system diseases, including PD [7, 8]. However, few studies have investigated the possible role of ceRNAs during LID.

In our previous study using transcriptome sequencing, we found a LID-related lncRNA, NONRATT023402.2, in a rat model [9]. However, the mechanism of how NONRATT023402.2 participates in LID has not been sufficiently verified. In the present study, we therefore characterized the ceRNA mechanism of NONRATT023402.2, and found that it participated in LID through the nerve growth factor receptor (*NGFR*) in a rat model.

## Results

### The expression of NONRATT023402.2 decreased in the early stage of LID, but increased after 6 weeks

**Fig. 1:**
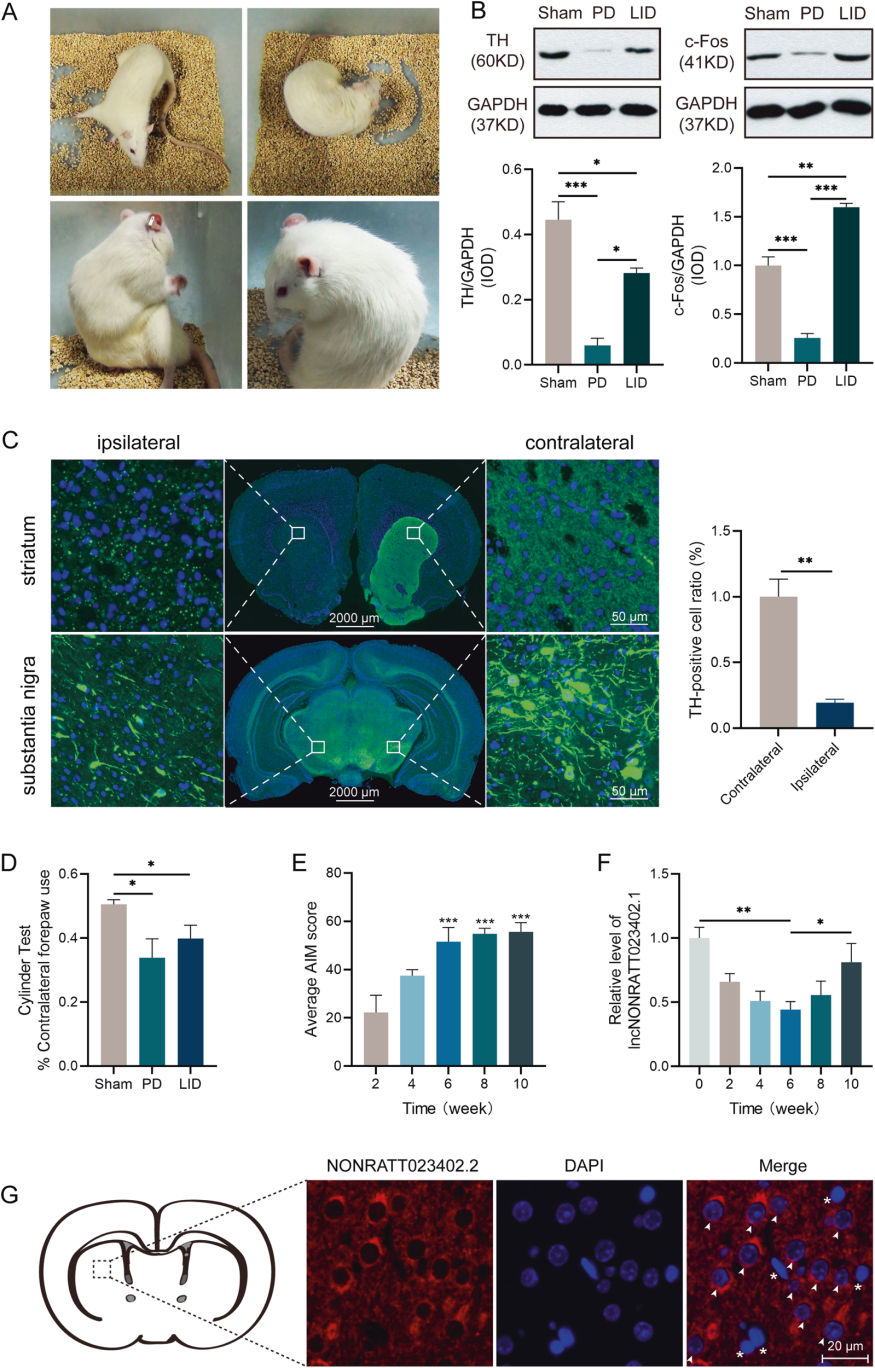
PD and LID model validation and NONRATT023402.2 and behavioral characteristics of long-term LID models. **A** Typical AIM included axial, forelimb, orolingual, and locomotive dyskinesia. **B** The protein level of TH and c-Fos in the right striatum of the Sham group, PD group, and LID group rats (*n* = 3). **C** Representative photomicrographs of TH immunofluorescence in coronal brain sections of the striatum and substantia nigra of rats subjected to 6-OHDA injection into the right striatum with L-DOPA administration. **D** Performance in the cylinder test of the Sham group, PD group, and LID group rats during a 3 week course (*n* = 5–6). **E** Average AIM scores of LID rats treated with L-DOPA for 2, 4, 6, 8, and 10 weeks on the day before being sacrificed (*n* = 4–5). **F** NONRATT023402.2 levels in the right striatum of LID rats treated with L-DOPA for 2, 4, 6, 8, and 10 weeks and PD rats (*n* = 4–5). **G** Fluorescence in situ hybridization labeling of NONRATT023402.2 in the striatum of rats. Arrows and asterisks indicate neurons and astrocytes, respectively. Data represent the mean ± SEM. *P < 0.05; **P < 0.01; ***P < 0.001.

The PD and LID models were validated by assessing behavioral and molecular changes in the second batch of rats. Striatal 6-OHDA-induced lesions resulted in a dramatic loss of dopaminergic neuron degeneration in PD and LID rats, which was shown using western blotting (Fig. 1B) and immunofluorescence (Fig. 1C) to measure TH. The rat model of LID was confirmed by assessing the expression of c-Fos in the striatum of LID rats. As expected, c-Fos protein levels were increased in LID groups relative to PD groups (Fig. 1B). Using immunofluorescence, it was found that the number of c-Fos positive cells in the striatum of LID rats was significantly more than those in PD rats (Fig. S1). Sham, PD, and LID groups treated with L-DOPA/benserazide for 3 weeks were used in the cylinder test to detect the use of forepaws. Compared with the sham group, the use of forepaws contralateral to the lesion side in the PD and LID groups decreased significantly to varying degrees (*P* < 0.05) (Fig. 1D).

Our previous study found that compared with non-treated PD rats, the expression of NONRATT023402.2 in rat striatum decreased significantly after 3 weeks of L-DOPA/benserazide administration [9]. In the present study, we extended the observation period to 10 weeks and recorded the behavior and NONRATT023402.2 expression level of LID rats every 2 weeks. The average AIM score of LID rats gradually increased with days of L-DOPA/benserazide treatment, and stabilized at the 6th week (Fig. 1E). However, the expression level of NONRATT023402.2 did not completely follow the same pattern. After L-DOPA/benserazide administration for 6 weeks, the expression of NONRATT023402.2 reached a low point, and then gradually increased (Fig. 1F). The changes of NONRATT023402.2 in long-term LID suggested that there may be a state of regulation and imbalance. RNA FISH analysis showed that NONRATT023402.2 was mainly localized in the cytoplasm of neurons throughout the brain, suggesting that it might act in a ceRNA manner (Fig. 1G).

### Overexpression of NONRATT023402.2 aggravated the AIM of LID rats

**Fig. 2:**
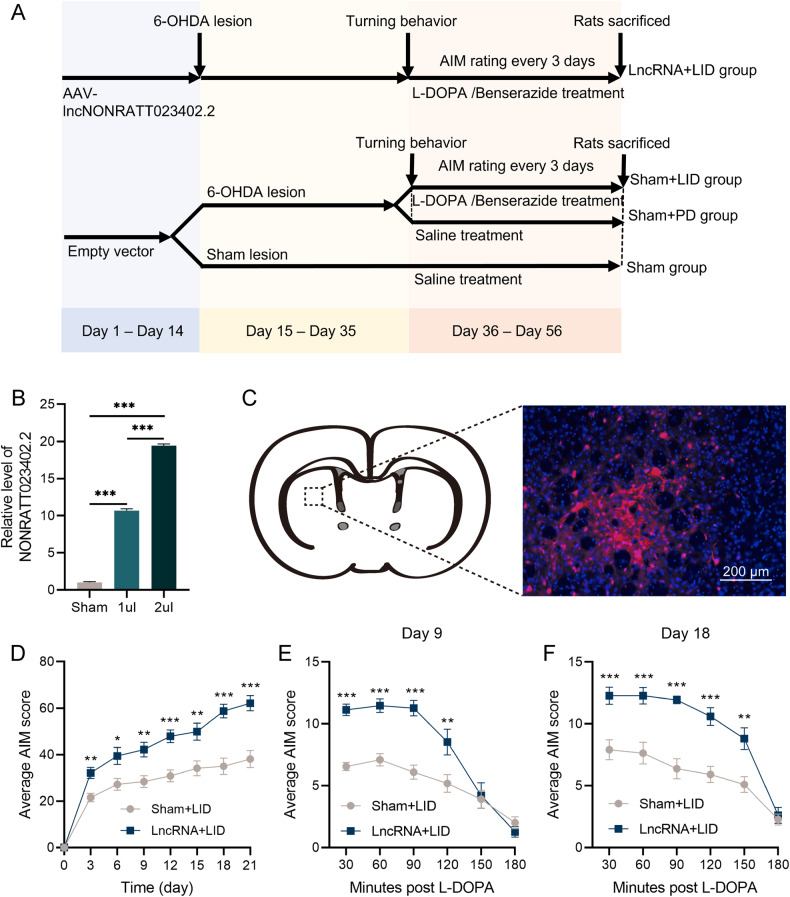
NONRATT023402.2 overexpression aggravates the AIM of LID rats. **A** Experimental timeline of the second batch. **B** Relative NONRATT023402.2 levels in the right striatum of rats injected with the empty AAV vector, 1 μL lncRNA-AAV, and 2 μL lncRNA-AAV (*n* = 3). **C** Red fluorescence in the right striatum of rats with the injection point as the center, as visualized by microscopy. **D** Time course of AIM development during the chronic L-DOPA treatment period of Sham + LID and LncRNA + LID group rats (*n* = 11 − 15). **E**, **F** Time course of the AIM score after a single injection of L-DOPA (treatment on days 9 and 18) of Sham + LID and LncRNA + LID group rats (*n* = 11–15). Data represent mean ± SEM. *P < 0.05; **P < 0.01; ***P < 0.001.

Before studying the effect of NONRATT023402.2 on LID, two doses of AAV-NONRATT023402.2 (1 μL per injection point and 2 μL per injection point) were used in a preliminary experiment (Fig. 2A). The NONRATT023402.2 level in the striatum of rats was detected by qRT-PCR, and it was found that NONRATT023402.2 in the 1 μL group and 2 μL group was about 10 times and 20 times that in the control group, respectively (Fig. 2B). This indicated that NONRATT023402.2 was efficiently transfected into rat brain tissue within this dose range. In addition, red fluorescence was observed in the right striatum of rats (2 μL group) with the injection point as the center, using a microscope, and indicating that the injection location was accurate (Fig. 2C).

In subsequent experiments, the virus was injected at a dose of 2 μL per injection point. The AIM scores showed significant differences between the sham + LID and LncRNA + LID groups in the second batch during 3 weeks of L-DOPA/benserazide treatment (*P* < 0.05) (Fig. 2D). The AIM score within 3 h after administration on the 9th (Fig. 2E) and 18th days (Fig. 2F) was separately analyzed. The AIM score gradually decreased after 90 min and disappeared after 3 h, which was consistent with drug metabolism. In the first 120−150 min, AIM scores of the LncRNA + LID group were significantly higher than those of the sham + LID group (*P* < 0.05). Together, these results indicated that overexpression of NONRATT023402.2 aggravated the AIM symptoms of LID rats.

### 316 DEGs were detected between LncRNA + LID and LID groups by sequencing

**Fig. 3:**
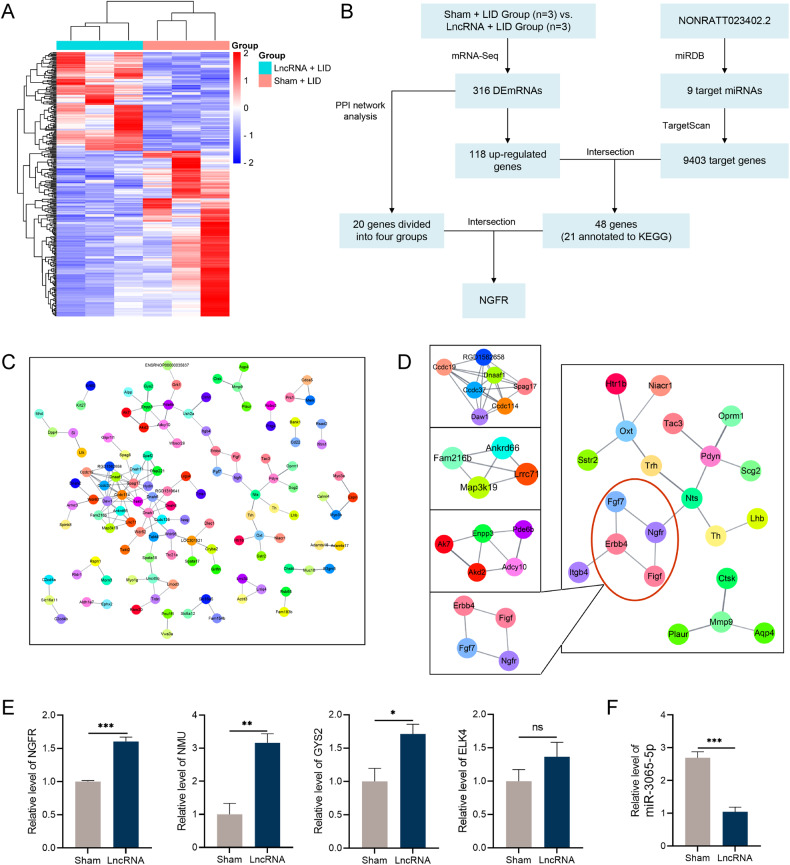
The mRNA expression signatures of Sham + LID and LncRNA + LID group rats. **A** Heat map of mRNA in the right striatum of LID rats with or without lncRNA-AAV injection (*n* = 3). **B** Schematic representation of the screening target genes process. The intersection of 118 up-regulated genes obtained by sequencing, and 9403 target genes predicted by the database were used, and then the *NGFR* was determined as the next research object according to the KEGG annotation and PPI network analysis. **C** PPI network of differentially-expressed genes. The nodes represent proteins, and the edges represent the interaction of proteins. **D** Four subgroups of the PPI network. **E** *NGFR*, *NMU*, *GYS2*, and *ELK4* expressions determined by qRT-PCR in the right striatum of the Sham + LID group, and LncRNA + LID group rats (*n* = 3–4). **F** The rno-miR-3065-5p expression in the right striatum of the Sham + LID group and LncRNA + LID group rats (*n* = 3–4). Data represent the mean ± SEM. *P < 0.05; **P < 0.01; ***P < 0.001.

Three LncRNA + LID rats and three sham + LID rats from the second batch were randomly selected for high throughput sequencing of their right striatum. A total of 30,961 genes were obtained by genome mapping. With a criterion of log_2_fold-change (FC) > 1.2 and *P* < 0.05, 316 DEGs were screened, of which 118 were up-regulated (Fig. 3A and S2A).

To further analyze the function of DEGs, GO terms and KEGG pathway annotation were used. The cAMP signaling pathway, PI3K-Akt signaling pathway, amyotrophic lateral sclerosis, MAPK signaling pathway, calcium signaling pathway, and Huntington’s disease were KEGG pathways with the most annotated DEGs (Fig. S2B). The cilium movement, motile cilium, and ATP-dependent microtubule motor activity were GO terms with the most DEGs enrichment of biological processes, cellular components, and molecular functions, respectively (Fig. S2C).

STRING was then used to analyze the PPI network, and the MCODE of Cytoscape was used to identify four closely connected protein groups in the network. We used the tissue filters tool to screen-out proteins related to the nervous system and found that one protein group met the requirements (Fig. 3B–D). *NGFR* was up regulated in this group of proteins.

### Bioinformatics analysis predicted the ceRNA mechanism of NONRATT023402.2

Rno-miR-350, rno-miR-29a-5p, rno-miR-509-3p, rno-miR-551b-5p, rno-miR-20a-3p, rno-miR-3065-5p, rno-miR-7a-1-3p, rno-let-7c-1-3p, and rno-miR-463-3p were identified as target miRNAs of NONRATT023402.2 using the miRDB database (Table S1).

According to the ceRNA mechanism, overexpressed lncRNA and target genes competitively bind to miRNAs, resulting in up-regulation of target genes. Therefore, we intersected the 118 upregulated genes with the predicted target genes to obtain 48 genes (Fig. 3B and Table S1).

**Fig. 4:**
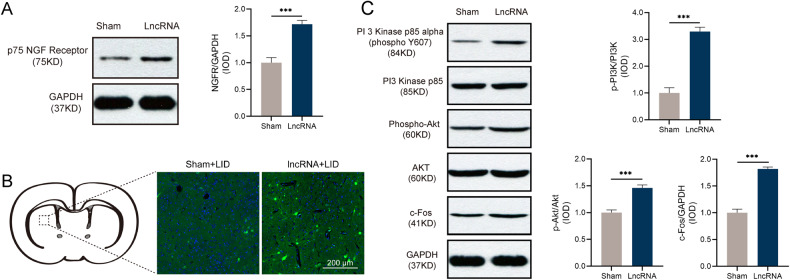
NONRATT023402.2 overexpression activated *NGFR* and PI3K/Akt signaling pathways. **A** Quantification of *NGFR* expression in the right striatum of Sham + LID and LncRNA + LID group rats (*n* = 4–5). The signal intensities of protein bands were normalized to that of GAPDH. **B** Immunofluorescence of *NGFR* in the right striatum of Sham + LID and LncRNA + LID group rats. **C** Quantification of p-PI3K, PI3K, p-Akt, Akt, and c-Fos expressions in the right striatum of Sham + LID and LncRNA + LID group rats (*n* = 4–5). The signal intensity of protein bands was normalized to that of GAPDH. Data represent the mean ± SEM. ***P < 0.001.

Combined with the function analysis and PPI network analysis of target genes, *NGFR* was identified as the gene of interest, and its expression level was determined using qRT-PCR. In addition, the expression levels of representative genes, *NMU*, *ELK4* and *GYS2*, were also detected. The expression levels of *NGFR*, *NMU*, and *GYS2* were increased in the brain tissues of NONRATT023402.2 overexpressing rats, which was consistent with the sequencing results (*P* < 0.05) (Fig. 3E). There was no significant difference in the expression levels of *ELK4* between the LncRNA + LID and sham + LID groups, but *ELK4* also showed an increasing trend in the LncRNA + LID group. We also used the same method to analyze rno-miR-3065-5p expression differences between the two groups, and found that rno-miR-3065-5p in the LncRNA + LID group was significantly down-regulated, which was consistent with the hypothesis (*P* < 0.05) (Fig. 3F). In addition, we determined the expression levels of NGFR protein and found that NGFR was significantly increased in LID rats overexpressing NONRATT023402.2 (Fig. 4A, B).

### Overexpression of NONRATT023402.2 activated the PI3K/ Akt pathway

Because it was confirmed that *NGFR* was the target gene of NONRATT023402.2, we specifically detected the downstream signaling pathways of the NGFR:PI3K/Akt signaling pathway. Compared with the sham + LID group, the protein levels of the p-Akt/Akt, p-PI3K/PI3K, and c-Fos in LncRNA + LID groups were significantly elevated (Fig. 4C), indicating that overexpression of NONRATT023402.2 in LID rats activated the PI3K/Akt signaling pathway.

### NONRATT023402.2 targeted rno-miR-3065-5p, and rno-miR-3065-5p targeted NGFR

**Fig. 5:**
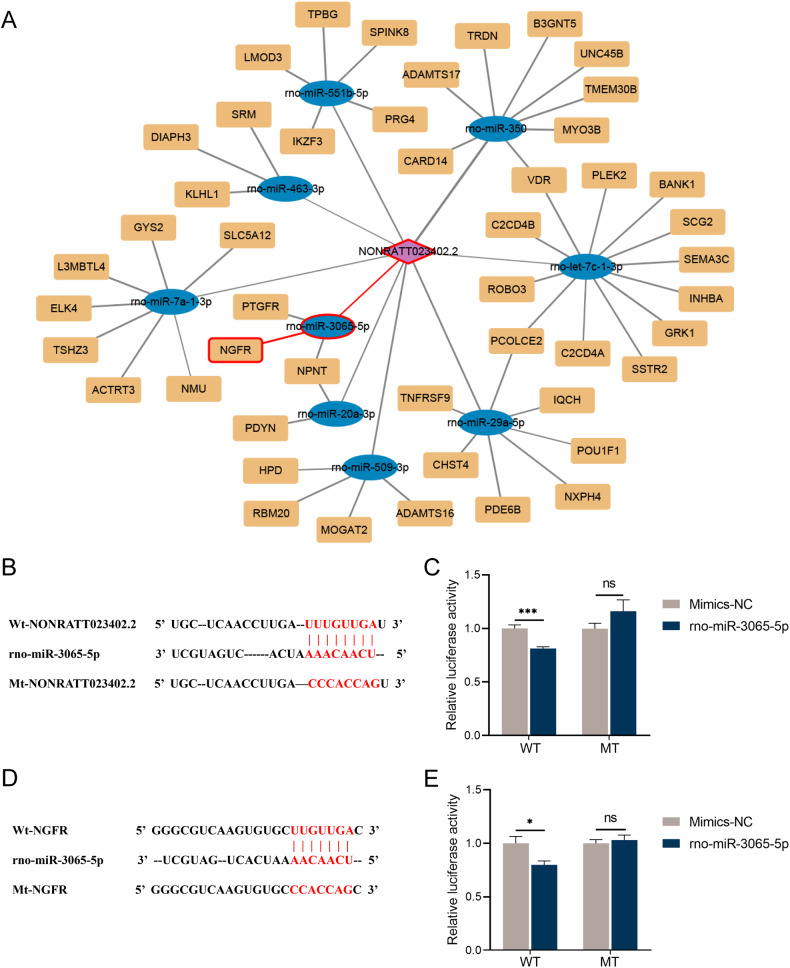
NONRATT023402.2 might function by sponging rno-miR-3065-5p, which targets the *NGFR*. **A** Construction of the ceRNA network demonstrated relationships among NONRATT023402.2, rno-miR-3065-5p, and *NGFR*. **B** Binding sites between NONRATT023402.2 and rno-miR-3065-5p, and the mutant sequence of NONRATT023402.2 based on the binding region. **C** Luciferase reporter assay for interactions between NONRATT023402.2 and rno-miR-3065-5p in HEK293FT cells. **D** Binding sites between rno-miR-3065-5p and *NGFR*, and the mutant sequence of *NGFR* based on the binding region. **E** Luciferase reporter assay for interactions between rno-miR-3065-5p and *NGFR* in HEK293FT cells. Data represent the mean ± SEM. *P < 0.05; **P < 0.01; ***P < 0.001.

The bioinformatics analysis revealed putative complementary sequences for NONRATT023402.2 and *NGFR* in rno-miR-3065-5p (Fig. 5A). The binding sites of NONRATT023402.2 and rno-miR-3065-5p were located at positions 1056–1063 of the 5′ end of the circRNAs, while the binding target of rno-miR-3065-5p with *NGFR* was located at positions 633–639 in the *NGFR* mRNA 3′-UTR region. The minimum free energy of NONRATT023402.2 and rno-miR-3065-5p was −17.2 kcal/mol, while the minimum free energy of rno-miR-3065-5p and *NGFR* was −24.9 kcal/mol.

The direct interactions between NONRATT023402.2 and rno-miR-3065-5p, rno-miR-3065-5p, and *NGFR* were then verified using luciferase reporter gene analysis. Co-transfection of the rno-miR-3065-5p mimic plasmid containing a sequence corresponding to position 1056–1063 in NONRATT023402.2 produced less luciferase activities (*P* < 0.05). Mutation of the binding sites abolished the repressive effect of rno-miR-3065-5p (Fig. 5B, C). Similarly, co-transfection of the rno-miR-3065-5p mimic plasmid containing a sequence of position 633–639 in the 3′-UTR of *NGFR* consistently produced less luciferase activity (*P* < 0.05), and mutation of the binding sites abolished the repressive effect of rno-miR-3065-5p (Fig. 5D, E).

### The expression of rno-miR-3065-5p increased in the early stage of LID, but decreased after 6 weeks

In order to further confirm the role of rno-miR-3065-5p and NGFR in LID, we measured the expression changes of rno-miR-3065-5p and NGFR in the striatum of LID rats within 10 weeks. As expected, rno-miR-3065-5p showed a trend of first increase and then decline, while the expression trend of NGFR was consistent with that of NONRATT023402.2, with a 6-week turning point of first decrease and then increase (Fig. S3).

### Knocking-down of rno-miR-3065-5p aggravated the AIM and activated the PI3K/ Akt pathway of LID rats

Before studying the effect of rno-miR-3065-5p on LID, 2ul of AAV-rno-miR-3065-5p were used in a preliminary experiment. The level of rno-miR-3065-5p in the striatum tissues of rats was determined by qRT-PCR. Compared to that in the control group, rno-miR-3065-5p expression was significantly reduced by AAV injection in the striatum (Fig. S4A, B). This indicated that AAV-rno-miR-3065-5p inhibitor was efficiently transfected into rat striatum tissue. The AIM scores showed significant differences between the sham and microRNA groups in the second batch during 3 weeks of L-DOPA/benserazide treatment (*P* < 0.05) (Fig. S4C). The AIM score within 3 h after administration on the 9th and 18th days was separately analyzed (Fig. S4D, E). The AIM score also gradually decreased after 90–120 min and disappeared after 3 h. In the first 120–150 min, AIM scores of the microRNA group were significantly higher than those of the sham group (*P* < 0.05). Consistent with overexpression of NONRATT023402.2, knocking-down of rno-miR-3065-5p also activated NGFR: PI3K/Akt signaling pathway. Compared with the sham group, the protein levels of the p-Akt/Akt, p-PI3K/PI3K, and c-Fos in, microRNA groups were significantly elevated (Fig. S4F, G). Thus, the above results indicated that inhibition of rno-miR-3065-5p aggravated the AIM symptoms through activated the PI3K/ Akt pathway of LID rats.

### Knocking-down NGFR reduced the AIM of LID rats in vivo

**Fig. 6:**
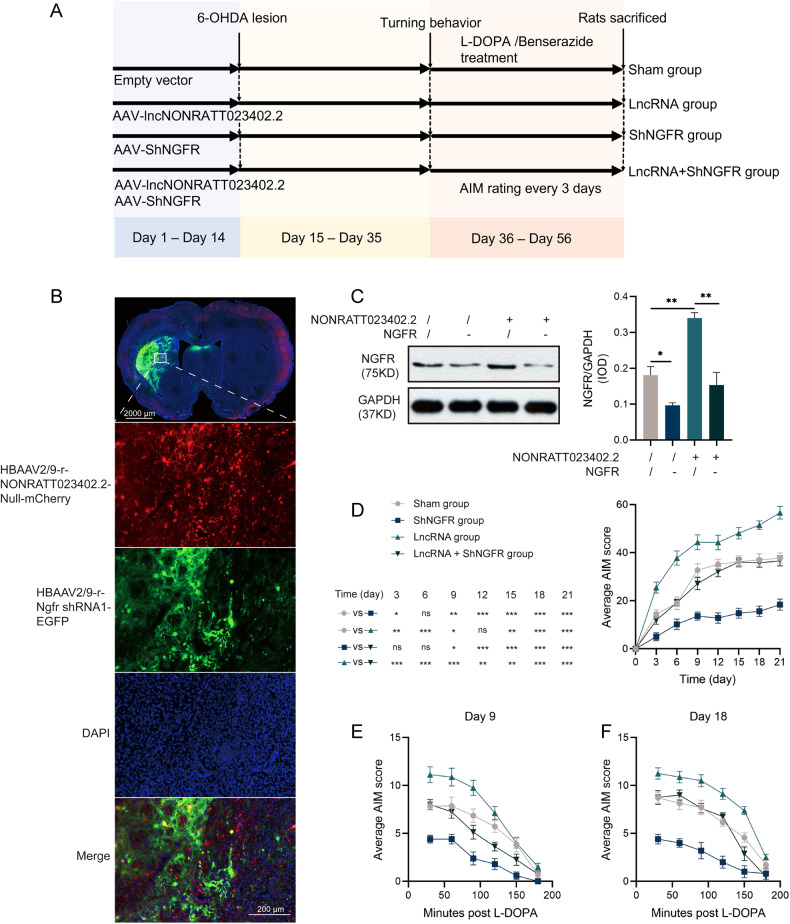
*NGFR* knockdown alleviates the AIM of LID rats. **A** Experimental timeline of the third batch. **B** Representative fluorescence images of the right striatum of rats with the injection point as the center, as visualized by microscopy. Cells overexpressing NONRATT023402.2 expressed red fluorescence, while cells with knock-downed *NGFR* expressed green fluorescence. Nuclei were counterstained with DAPI (blue). **C** The protein levels of *NGFR* in the right striatum of Sham, ShNGFR, LncRNA, and LncRNA + ShNGFR group rats (*n* = 3). **D** Time course of AIM development during the chronic L-DOPA treatment period of Sham, ShNGFR, LncRNA, and LncRNA + ShNGFR rats (*n* = 5–8). **E**, **F** Time course of AIM score after a single injection of L-DOPA (treatment on days 9 and 18) of Sham, ShNGFR, LncRNA, and LncRNA + ShNGFR rats (*n* = 5−8). Data represent the mean ± SEM. *P < 0.05; **P < 0.01; ***P < 0.001.

The four groups of LID rats (sham, LncRNA, ShNGFR, and LncRNA + ShNGFR groups) were used for subsequent studies (Fig. 6A). Depending on the grouping, NONRATT023402.2-AAV, ShNGFR-AAV, or empty vector were injected at a dose of 2 μL per injection point. The method of LID model preparation and behavioral observation was the same as the second batch. Regarding the samples from rats in the LncRNA + ShNGFR group, microscopic observations showed that both NONRATT023402.2-AAV and ShNGFR-AAV were successfully transfected (Fig. 6B). In addition, the NGFR protein was significantly reduced in the striatum of rats injected with ShNGFR-AAV (*P* < 0.05), indicating that *NGFR* knockdown by ShNGFR-AAV was feasible (Fig. 6C).

Among the four groups, the AIM score of the sham group was the highest, the AIM score of the ShNGFR group was the lowest, and the AIM scores of the other two groups were similar and between the highest and lowest scores (Fig. 6D). There was a significant difference in AIM scores between the sham and LncRNA groups, except on the 12th day (*P* < 0.05). From the 9th day, the AIM score of the LncRNA + ShNGFR group was significantly higher than that of the ShNGFR group (*P* < 0.05). These results were the same as those of the second batch of experiments, which further confirmed that overexpression of NONRATT023402.2 aggravated AIM symptoms of LID rats. Except for the 6th day, there were significant differences in AIM scores between the sham and ShNGFR groups (*P* < 0.05). During the whole experimental process, the AIM score of the LncRNA group was significantly higher than that of the LncRNA + ShNGFR group (*P* < 0.05). These results suggested that down-regulation of *NGFR* alleviated AIM symptoms, which was consistent with the results of Liu et al. The AIM score within 3 h after administration on the 9th and 18th days showed the same drug metabolism pattern in each group, and the differences among each group were consistent with the overall AIM score (Fig. 6E, F).

After these behavioral observations were assessed, the four groups of rats were sacrificed and the expressions of NONRATT023402.2 and *NGFR* in the striatum were detected by qRT-PCR. By comparing the expression of NONRATT023402.2 in each group, we found that NONRATT023402.2-AAV could significantly improve its expression (*P* < 0.01), which was consistent with the results of the second batch.

**Fig. 7:**
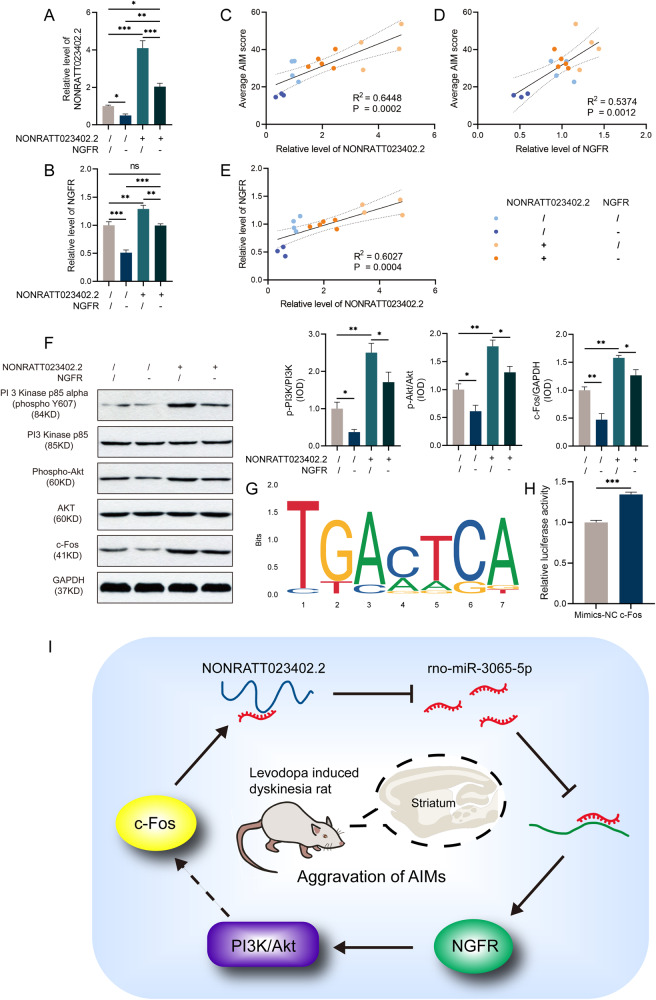
*NGFR* knockdown inhibits the PI3K/Akt signaling pathway and c-Fos promotes NONRATT023402.2 expression. **A** NONRATT023402.2 expression determined by qRT-PCR in the right striatum of Sham, ShNGFR, LncRNA, and LncRNA + ShNGFR rats (*n* = 3−5). **B** *NGFR* expression determined by qRT-PCR in the right striatum of Sham, ShNGFR, LncRNA, and LncRNA + ShNGFR rats (*n* = 3). **C** The AIM score was positively correlated with the NONRATT023402.2 expression level (*n* = 16). **D** The AIM score was positively correlated with the *NGFR* expression level (*n* = 16). **E** The NONRATT023402.2 expression level was positively correlated with the *NGFR* expression level (*n* = 16). **F** Quantification of p-PI3K, PI3K, p-Akt, Akt, and c-Fos expressions in the right striatum of Sham, ShNGFR, LncRNA, and LncRNA + ShNGFR rats (*n* = 3). The signal intensities of protein bands were normalized to that of GAPDH. **G** Representative sequence of c-Fos binding specificity queried from JASPAR (identifier MA0099.2). **H** Luciferase reporter assay for interactions between c-Fos and NONRATT023402.2 in HEK293FT cells. **I** A working model showing the mechanism of the NONRATT023402.2/ miR-3065-5p/ *NGFR* axis in the striatum of LID rats. NONRATT023402.2 targets miR-3065-5p/ *NGFR* axis as a ceRNA and activates the PI3K/Akt signaling pathway and c-Fos to aggravate AIM symptoms. The c-Fos, as a transcription factor, in turn promotes NONRATT023402.2 expression, forming a positive feedback loop in the rat model of LID. Data represent the mean ± SEM. *P < 0.05; **P < 0.01; ***P < 0.001.

However, the level of NONRATT023402.2 in the ShNGFR +LID group was significantly lower than that in the sham +LID group, and the level of NONRATT023402.2 in the LncRNA + ShNGFR +LID group was significantly lower than that in the LncRNA +LID group (*P* < 0.01) (Fig. 7A). This suggested that *NGFR* had a regulatory effect on NONRATT023402.2, forming a positive feedback regulation loop. The expression of *NGFR* could be the result of the combined action of NONRATT023402.2 and *NGFR* intervention. The expression of *NGFR* in the ShNGFR group was significantly lower than that in the sham and LncRNA + ShNGFR groups (*P* < 0.01). In contrast, its expression in LncRNA group was significantly higher than that in these two groups (*P* < 0.01), which was consistent with the AIM scores in each group (Fig. 7B). We next conducted correlation analysis to identify the relationship between them. The expression levels of NONRATT023402.2 and *NGFR* were significantly positively correlated with AIM scores (Fig. 7C, D), and the expression levels of NONRATT023402.2 and *NGFR* were significantly positively correlated with these results (Fig. 7E).

### Knockdown of NGFR inhibited the PI3K-Akt signaling pathway in vivo and in vitro

Consistent with the results of the second batch, the expressions of p-PI3K/PI3K, p-Akt/Akt, and c-Fos in the LncRNA group were significantly increased, when compared with the sham group (*P* < 0.01). These proteins were significantly decreased after *NGFR* knockdown, regardless of whether lncRNA-AAV was present (*P* < 0.05) (Fig. 7F). This supported the hypothesis that NONRATT023402.2 regulated the PI3K-Akt signaling pathway via *NGFR*. To verify the specificity of AAV-shNGFR and prevent off-target effect, we use different sequences of siRNA to knockdown the level of NGRF in primary striatal neurons of SD rats. The transfection efficiency was examined by qRT-PCR and Western blot. Compared with control group, si-NGFR#1, si-NGFR#2, si-NGFR#3 and si-NGFR#AAV (siRNA sequence from shNGFR sequence) significantly inhibitor the level of NGFR (Fig. S5A). Consistent with the results in vivo, the protein level of p-PI3K/PI3K, p-Akt/Akt, and c-Fos in the si-NGFR groups were significantly decreased (Fig. S5B, C). In addition, the level of c-Fos suggested that c-Fos may play a role as a downstream molecule of *NGFR*, which supported the hypothesis that c-Fos regulated the expression of NONRATT023402.2.

### C-Fos facilitated the expression of NONRATT023402.2 by binding to its promotor region

To understand how *NGFR* regulated the expression of NONRATT023402.2, we searched the transcription factors of NONRATT023402.2 in the JASPAR and PROMO databases and found that c-Fos has a high affinity for binding to the promotor region of NONRATT023402.2 (Fig. 7G, S6). Western blot analysis showed that knockdown of *NGFR* resulted in a decrease in c-Fos levels (Fig. 7F). However, c-Fos levels were decreased in NONRATT023402.2 overexpressing rats, suggesting that NONRATT023402.2 may have affected c-Fos expression in other ways besides through the *NGFR*.

We then performed dual luciferase assays to determine whether c-Fos was an activator or a repressor of NONRATT023402.2 transcription. The relative luciferase activity was higher in the presence of the effector than with the control, suggesting that c-Fos directly bound to the NONRATT023402.2 promotor, and acted as a transcriptional activator (Fig. 7H).

## Discussion

The introduction of oral L-DOPA as a treatment to control the motor symptoms of PD patients has been a great success in modern neuroscience. However, LID occurs in up to 80% of patients with PD after a few years of chronic treatment, so it has become one of the most clinically challenging factors in long-term management of PD patients [10]. Accumulating evidence has indicated the important roles of lncRNAs in different diseases, including PD [11]. NEAT1 plays a protective role in PD by increasing the stabilization of PINK1 [12]. LncRNA HOTAIR promotes PD progression by regulating rno-miR-126-5p and RAB3IP in a ceRNA-dependent manner [13]. MALAT1 lncRNA has been reported to promote apoptosis in dopaminergic neurons by sponging rno-miR-124 in PD [14]. LncRNA MALAT1 has also been reported to affect the CREB pathway, which contributes to cognitive deficits occurring during neurodegenerative diseases [15]. We believe that lncRNAs also play an important role in LID. The present research is an extended study on differentially-expressed NONRATT023402.2 in LID rats, with the aim of clarifying the mechanism of lncRNA in LID.

NONRATT023402.2 is highly conserved in many species, including humans [9], which demonstrates the importance of its role, and facilitates the transfer of future research to clinical applications. We found that the expression of NONRATT023402.2 in LID rats gradually decreased in the first 6 weeks, but then increased. Due to the increased mortality of rats caused by prolonged AIM, data from longer studies were missing, and valid data were only recorded until week 10. Considering the aggregation of AIM in NONRATT023402.2 overexpressing rats, we believe that this lncRNA may play a role in promoting LID, and that the early reduction of expression may be a self-protection mechanism. With disease progression, when the self-protection mechanism is dysregulated, the expression of this NONRATT023402.2 may increase. Based on the qPT-PCR results, the expression of NONRATT023402.2 in LID rats injected with lncRNA-AAV at week 3 was much higher than that in LID rats without lncRNA-AAV injection at week 10. In terms of AIM scores, lncRNA-AAV injection accelerated the time of AIM reaching a high value plateau; the AIM score of LncRNA rats at week 3 was similar to the AIM score of LID rats at week 6, 8, and 10 in the first batch.

*NGFR* is highly conserved between species and encodes the p75 neurotrophin receptor (p75NTR), which binds all members of the neurotrophin family. The p75NTR can induce neuronal cell proliferation, migration, differentiation, survival, apoptosis, and synaptic plasticity via different signal transduction pathways, and exert multiple biological effects. The p75NTR is involved in psychiatric disorders [16], Alzheimer’s disease [17, 18] depression [19], anxiety [20], ischemia [21], and other neurological or psychiatric diseases. Studies have shown a strong link between *NGFR* and Huntington’s disease (HD), which is an inherited neurological disorder. Dysfunctional *NGFR* signaling contributes to dendroid spine loss and plasticity deficits that occur in HD mouse models [22]. The p75NTR levels are elevated in the stratum and hippocampus of HD patients, and downregulation of p75NTR expression can delay HD progression in mouse models [23, 24]. There are many similarities between LID and HD in their manifestations, molecular characteristics, and treatments. The functional annotation analysis of DEGs in this study also suggested that many DEGs are involved in the pathogenesis of HD (Fig. S2B). It has been reported that *NGFR* was expressed in the nigral dopamine neurons and may play important roles in neuronal cell survival or excitotoxic degeneration of dopamine neurons in the substantia nigra during the pathogenesis of PD [25–27]. A study on the role of p75NTR in LID found that knocking-down striatal p75NTR prevented the development of LID and increased striatal structural plasticity in rat models. In contrast, striatal p75NTR overexpression exacerbated LID and facilitated the loss of striatal dendritic spines in rats [28]. These results are consistent with the present study and support the idea that *NGFR* is involved in LID.

PI3K/Akt is one of the most important signaling pathways involved in PD [29, 30], but its role in LID has been rarely reported. The PI3K/Akt signaling pathway can be activated by *NGFR*, and there is an interaction between the PI3K/Akt and MAPK signaling pathways, which are important pathways related to LID. This study found that NONRATT023402.2 activated the PI3K/Akt signaling pathway by upregulating *NGFR* and affecting the severity of LID. These results extended our current understanding of the PI3K/Akt signaling pathway in this process.

According to the NONRATT023402.2/ rno-miR-3065-5p/ *NGFR* axis model, the phenomenon of NONRATT023402.2 expression decreased by ShNGFR-AAV injection cannot be explained. However, we were able to identify a possible mechanism of *NGFR* regulating NONRATT023402.2: transcription factor c-Fos. The c-Fos is a major member of the Fos transcription factor family. Fos family members and Jun family members form heterodimers through leucine zipper structures and bind to the promoters of target genes to promote their expressions. The c-Fos gene is a type of immediate-early gene, which can transiently and rapidly be activated in response to a wide variety of stimuli. Studies have shown that c-Fos was highly expressed in striatum of LID animals, and could be used as a hallmark for LID [31]. L-DOPA and dopamine agonist treatment induces striatal c-Fos expression in striatum, and inhibition of c-Fos activation attenuates LID [32, 33]. A previous study reported that transgenic overexpression of ΔFosB in the striatum of Parkinsonian macaque induced LID-like behavioral and pathological changes [34]. The present study found that the expression level of c-Fos was affected by *NGFR* in LID rats, just like the PI3K/Akt signaling pathway. Previous studies have also confirmed the regulatory effect of two cross-talk signaling pathways, PI3K/Akt and MAPK, on downstream c-Fos in different diseases [35–37]. In the present study, we found a novel mechanism by which c-Fos participated in LID, involving activation of NONRATT023402.2 transcription to achieve positive feedback regulation of the NONRATT023402.2/rno-miR-3065-5p/ *NGFR* axis.

LncRNAs have multiple mechanisms of action, such as chromatin modification, microRNA sequestration by sponging, mRNA stabilization, splicing modulation, and degradation of target mRNAs. In the present study, we only discussed the possibility of NONRATT023402.2 acting as a molecular sponge in LID. However, NONRATT023402.2 may participate in the regulation of LID through various mechanisms, which should be tested in future studies.

It is important to note that the effect of the ceRNA depends on its concentration [6, 38]. In the present study, overexpression of NONRATT023402.2 or knockdown of *NGFR* did not simulate a disease state, but instead was a method to study their relationship and affects on LID. The expression changes caused by external intervention under experimental conditions did not occur during the actual disease course, but the behavioral changes and downstream molecular changes presented in this study were sufficient to provide strong support for our hypothesis.

In conclusion, for the first time, our study demonstrated that NONRATT023402.2 impaired rno-miR-3065-5p activity through sequestration, thereby upregulating *NGFR* expression, and further activating the downstream PI3K/Akt signaling pathway and c-Fos protein, which aggravated the AIMs of PD rats treated with L-DOPA. The expression of NONRATT023402.2 was then up-regulated by c-Fos, forming a positive feedback loop (Fig. 7I). Taken together, these findings provide novel insight into a possible treatment for LID.

## Materials and methods

### Animals

Male Sprague-Dawley rats weighing 200–250 g were obtained from Vital River Experimental Animal Technology (Beijing, China) and were housed in a temperature-controlled room (23 °C) on a 12-h light/dark cycle with food and water available *ad libitum*. All animal experiments were conducted according to the Chinese Animal Welfare Act, Beijing Guidelines for the Care and Use of Laboratory Animals and Guidance for Animal Experimentation of Capital Medical University. The study protocol was approved by the Ethics Committee of Beijing Neurosurgical Institute, Capital Medical University (protocol no. AEEI-2018-200).

### Adeno-associated virus (AAV vector)

An adeno-associated virus (AAV) vector was used to genetically overexpress NONRATT023402.2 (AAV2/9-NONRATT023402.2-mCherry, 1.5 × 10^12^ vg/mL), inhibitor rno-miR-3065-5p (AAV2-rno-miR-3065-5p-EGFP, 1.6 × 10^12^ vg/mL), or silence *NGFR* (AAV2/9-*NGFR* shRNA- EGFP, 6.3 × 10^12^ vg/mL). The empty AAV vector coding mCherry was used as the control. The shRNA sequence targeting *NGFR* was 5′-GCAGATGTGCCTATGGCTACT-3′ and the scrambled sequence was 5′-GAAGCAACTCGTCTGGACAGT-3′, which were based on a previous study [28]. The AAV vector was constructed by HanBio Biotechnology (Shanghai, China). The inhibitor sequence of rno-miR-3065-5p was 5′-AGCATCAGTGATTTTGTTGA-3′ and the scrambled sequence was 5′-TTCTCCGAACGTGTCACGT-3′. The AAV vector was constructed by Genepharma (Shanghai, China).

### Surgical procedures and pharmacology

The intracerebral microinjections were performed via stereotaxic injections, as previously described [9]. Briefly, rats were anesthetized with 2–3% isoflurane using an animal anesthesia ventilator system (RWD Life Science, Shenzhen, China) before stereotactic surgery. A total of 4 μL AAV was infused into the right striatum through a microsyringe (Hamilton, Reno, NV, USA) at a speed of 0.2 μL/min. The needle remained in place for an additional 5 min before it was slowly retracted. The coordinates for the two sites of striatum were anteroposterior (AP) = 0.5 mm, mediolateral (ML) = −3.0 mm relative to bregma, and dorsoventral (DV) = −4.5/−6.0 mm from the skull surface. Each site was injected with 2 μL AAV.

Two weeks later, 5 mg/mL 6-hydroxydopamine (6-OHDA) (Sigma-Aldrich, St. Louis, MO, USA) was injected into the medial forebrain bundle (from bregma: AP = −4.3 mm, ML = −1.6 mm, and DV = −8.4 mm from skull surface) and the substantia nigra pars compacta (from bregma: AP = −4.8 mm, ML = −1.7 mm, and DV = −8.0 mm from the skull surface) to achieve a full nigrostriatal lesion. Each site received 2 μL with an injection rate of 0.5 μL/min. The sham lesion group received the same dose of saline instead of 6-OHDA. Other operations were the same as the above AAV vector injection process.

To assess the successful establishment of a PD model, rats were injected subcutaneously with 0.5 mg/kg apomorphine (Sigma-Aldrich) 3 weeks after the 6-OHDA-induced lesion, with the turning behavior recorded. Rats displaying more than 7 full contralateral turns/min during the 30 min period after the injection of apomorphine were selected for L-DOPA administration.

Rats received a single daily intraperitoneal injection of L-DOPA (12 mg/kg; Sigma-Aldrich) combined with benserazide (6 mg/kg; Sigma-Aldrich). The sham LID group rats received single daily injections of the same volume of saline.

Three batches of animal experiments were conducted. The first batch was divided into the 0 week (PD) group (*n* = 4), 2 weeks group (*n* = 4), 4 weeks group (*n* = 4), 6 weeks group (*n* = 5), 8 weeks group (*n* = 4), and 10 weeks group (*n* = 5) according to the duration of L-DOPA treatments. The second batch included the LncRNA + LID group (*n* = 15), sham + LID group (*n* = 11), sham + PD group (*n* = 5), and sham group (*n* = 6). The LID group was injected with empty vector containing a fluorescein label. The third batch included the empty vector of the NONRATT023402.2/ShNGFR (sham) group (n = 7), AAV-NONRATT023402.2 and the empty vector of the ShNGFR (LncRNA) group (n = 5), AAV-ShNGFR and the empty vector of the NONRATT023402.2 (ShNGFR) group (*n* = 8), and the AAV-NONRATT023402.2/ShNGFR (LncRNA + ShNGFR) group (*n* = 8). The fourth batch included the empty vector of the rno-miR-3065-5p (sham) group and the AAV-rno-miR-3065-5p inhibitor (microRNA) group (*n* = 7).

### Behavioral testing

The cylinder test was used to evaluate forepaw use contralateral to the lesion side and to assess the success of modeling [39, 40]. Each rat was placed into a transparent cylinder (20 cm diameter and 30 cm height) and recorded for 5 min. The number of weight-bearing contacts made on the cylinder wall with the left, right, or both forepaws was recorded. Contralateral forepaw use was calculated according to the following equation: [(the number of contralateral forepaw movements)/(total number of forepaw movements) + (1/2) both forepaw movements] ×100% [41].

To assess dyskinesia behavior, rats were observed for AIM as described previously [9, 42]. In brief, the rats were monitored for 1 min every 30 min over a period of 3 h, immediately after L-DOPA administration for signs of axial, forelimb, orolingual dyskinesia, and locomotive activities (Fig. 1A). The scale of each sign was rated from 0 to 4 based on duration and severity. Total AIM scores were calculated as the sum of the score per observation point.

### Primary striatal neuronal cultures

Rat striatum tissues were extracted from fetal SD rats (embryonic day 17) under sterile conditions. The striatum tissues were digested for 10 min in Dulbecco’s modified Eagle’s medium (DMEM, Thermo Scientific, MA, United States) containing 0.02% papain at 37°C and the tissues were gently triturated for 15–20 times, followed with 8 min centrifugation. Then cells were plated onto flasks or 6-wells plates precoated with poly-D-lysine (Sigma-Aldrich, St. Louis, United States) at a density of 5 × 10^5^ cells/ml. The plating medium included DMEM, 10% fetal bovine serum (FBS), 5% horse serum, 1 mM L-glutamine. After 4 h, discard the plating medium and use neurobasal medium (Thermo Scientific) supplemented with 2% B27 (Thermo Scientific). On the 7th day, cells were used for processing and experiments.

### RNA interference

On the basis of the manufacturer’s protocol, cells were transfected with siRNA using LipofectamineTM 3000 reagent (Thermo Scientific). The knockdown efficiency was determined by RT-qPCR 24 h after transfection. siRNA sequences are as follows (Table 1).

**Table 1:** siRNA sequences.

Gene	Sense (5′-3′)	Antisense (5′-3′)
NC	UUCUCCGAACGUGUCACGUTT	ACGUGACACGUUGGGAGAATT
si-NGFR#1	GGUGCCAAGGAGACAUGUUTT	AACAUGUCUCCUUGGCACCTT
si-NGFR#2	GGGCACAUACUCAGACGAATT	UUCGUCUGAGUAUGUGCCCTT
si-NGFR#3	CCAGUACAGUGGCGGAUAUTT	AUAUCCGCCACUGUACUGGTT
si-NGFR#4	GGGUUACCAGCCUGAACAUTT	AUGUUCAGGCUGGUAACCCTT

### RNA extraction and sequencing

Rats were deeply anesthetized with isoflurane and decapitated. The striatum was separated using a microdissection procedure. Total RNA was isolated from the right striatum of rats using a TransZol Up Plus RNA Kit (Cat#ER501-01; TransGen Biotech, Beijing, China) following the manufacture’s instructions, and checked for an RNA integrity number to inspect RNA integrity using an Agilent Bioanalyzer 2100 (Agilent Technologies, Santa Clara, CA, US). Qualified total RNA was further purified by a RNAClean XP Kit (Cat# A63987; Beckman Coulter, Brea, CA, USA) and RNase-Free DNase Set (Cat#79254; Qiagen, Hilden, Germany).

The cluster was generated by cBot with the library diluted to 10 pM and then sequenced on an Illumina HiSeq X-ten system (Illumina, San Diego, CA, USA). Library construction and sequencing were performed by Shanghai Biotechnology (Shanghai, China). Raw reads were preprocessed by filtering-out rRNA reads, sequencing adapters, short-fragment reads, and other low quality reads. HISAT2 (version 2.0.4) [43] was used to map the clean reads to the Rnor6.0 reference genome. To quantify the mRNAs, their expressions were determined as fragments per kilobase of transcript per million mapped reads using StingTie (version 1.3.1) [44]. Differentially expressed genes (DEGs) were identified using the edgeR package [45] with a threshold log_2_fold-change (FC) > 1.2 and *P* < 0.05. Gene Ontology (GO) and Kyoto Encyclopedia of Genes and Genomes (KEGG) terms were annotated to the DEGs.

### Prediction of target miRNAs and target genes, and gene network construction

The potential target miRNAs of NONRATT023402.2 were predicted using the miRDB database [46], and the target genes of miRNAs were predicted using the TargetScan database [47]. The predicted target genes were intersected with upregulated DEGs to narrow the scope of the study.

The lncRNA-miRNA-mRNA ceRNA network was constructed based on the relationships between lncRNAs, using predicted target miRNAs and target mRNAs in Cytoscape (version 3.9.1). Among the target genes, the protein-protein interaction (PPI) network was obtained from the STRING database with a confidence > 0.2, with Cytoscape used for visualization. The module of the PPI network was analyzed using the MCODE plug [48].

### Dual luciferase reporter assays

The interactions between NONRATT023402.2 and rno-miR-3065-5p, rno-miR-3065-5p, *NGFR*, and c-Fos, and the promotor region of NONRATT023402.2 were verified using the dual-luciferase reporter assay. The luciferase reporter plasmids, pMIR-REPORT and pGL4.74 (Syngenbio, Beijing, China), encoding firefly luciferase (hluc+) and Renilla luciferase (hRluc), respectively, were used for all assays. The sequences of NONRATT023402.2, rno-miR-3065-5p, *NGFR*, and the promotor region of NONRATT023402.2 were obtained from the National Center for Biotechnology Information, and their binding sites were predicted using RNAhybrid (version 2.2).

Wild-type and mutant sequence fragments of NONRATT023402.2 and *NGFR* were inserted into their respective plasmids. HEK293FT cells were seeded into 96-well plates, and 100 μL of transfection solution containing 0.5 µL Lipofectamine 3000 (Invitrogen, Carlsbad, CA, USA) and 25 ng reporter plasmids with 50 nM rno-miR-3065-5p mimic or 50 nM mimic control were added, respectively. The cells were then washed and harvested 24 h after transfection. Luciferase activities were measured using the Dual Luciferase Reporter Assay Kit (Promega, Milwaukee, WI, USA) and Centro XS (Berthold Technologies, Bad Wildbad, Germany). No mutant group was used in the assay of the c-Fos and NONRATT023402.2 promoters. The experiments were then repeated six times.

### Quantitative real-time PCR

Total RNA was extracted using the Total RNA Extraction Kit (DNase I) (Cat#GPQ1801; GenePool) and reverse transcription was performed using the lncRNA cDNA Synthesis Kit (Cat#GPQ1806; GenePool) according to the manufacturer’s instructions. The qRT-PCR was performed in 20 μL reaction tubes containing 10 μL FastSYBR Mixture, 0.4 μL of each primer (10 μM), 2 μL cDNA template, and 7.2 μL dH_2_O using a BIOER LineGene 9600Plus instrument (Bioer Technology, Hangzhou, China) under the following conditions: 95 °C for 10 min, followed by 40 cycles of 95 °C for 15 s, and 60 °C for 60 s. The forward and reverse primer sequences are listed in Table 2. The relative expression levels were calculated using the 2^−ΔΔCT^ method after normalization with the reference control.

**Table 2:** Primers used in analysis of genes expression by qRT-PCR.

Primer name	Forward sequence	Reverse sequence
NONRATT023402.2	GGCTATTCATACAAAGTGGCAGTT	CGCTGAGTCTCGTGAGTCTG
miR-3065-5p	TCAACAAAATCACTGATGCTTTTT	
NGFR	GAGGGCACATACTCAGACGA	CTCTTCGCATTCAGCATCAG
NMU	CCAGAAGCCTCAGGAGCAA	GCACGACAGACGACACAAC
GYS2	GTTACACGCCAGCCGAATG	CCAGGTATCTCCAGTCCAGAAG
ELK4	CTCCTCCAGTTCCTTCCATACC	GGCTCCAGTGACAAGTTCTCT
GAPDH	TGGAGTCTACTGGCGTCTT	TGTCATATTTCTCGTGGTTCA
U6	CTCGCTTCGGCAGCACA	AACGCTTCACGAATTTGCGT

### Western blot analysis

Western blot analysis was performed as previously described [9] using the following primary antibodies: TH antibody (ab112, 1:200), c-Fos antibody (ab7963, 1:500), p75 NGF receptor antibody (ab52987, 1:1000), and PI3 kinase p85 alpha (phosphor Y607) antibody (ab182651, 1:500) (all from Abcam, Cambridge, MA, USA); PI3 kinase p85 (19H8) antibody (#4257; 1:500), AKT antibody (#9272; 1:1000), and phospho-Akt (Ser473) antibody (#9271, 1:1000) (all from Cell Signaling Technology, Danvers, MA, USA). GAPDH antibody (ab181602; 1:3000; Abcam) was used for the loading control. Protein band density was quantified using Quantity One software (version 4.6.2, Bio-Rad, Hercules, CA, USA).

### Immunofluorescence (IF)

Rats were anesthetized and transcardially perfused with 0.9% saline solution followed by 4% cold paraformaldehyde (PFA). Harvested rat brain tissues were fixed in 4% PFA and embedded in paraffin. The specimens (4 μm thick) were dried, washed, permeabilized, blocked in 5% goat serum, and incubated overnight at 4 °C with tyrosine hydroxylase (TH) antibody (ab112; 1:700), c-Fos antibody (ab7963; 1:50), or p75 NGF receptor antibody (ab52987; 1:100) (all from Abcam) and then incubated with the appropriate fluorochrome-conjugated secondary antibodies. Sections were mounted with medium containing diamidino-2-phenylindole (DAPI; to stain nuclei) (Vector Laboratories, Burlingame, CA, USA). The images were analyzed using Pannoramic Viewer software (3D HISTECH, Budapest, Hungary).

### RNA fluorescence in situ hybridization (FISH)

FISH was performed to detect the subcellular location of NONRATT023402.2. Brain sections were digested in a pepsin solution, fixed in formaldehyde, and dehydrated by gradient ethanol solutions. The sections were then incubated with a digoxin (DIG)-labeled probe (5′-DIG-AGTAACGCTGAGTCTCGTGAGTCTGGTTCCAT-DIG-3′), followed by incubation with a DyLight 594-conjugated IgG fraction (ab96873; Abcam) coupled with a monoclonal mouse DIG antibody (ab116590; Abcam;). Nuclei were then counterstained with DAPI.

### Statistical analysis

Statistical analyses were performed using SPSS statistical software for Windows, version 19.0.0 (SPSS, Chicago, IL, USA) and Prism 9.0.0 (GraphPad, La Jolla, CA, USA) software. Prior to significance testing, normal distribution and homogeneity of variances were confirmed by Shapiro-Wilk test and Brown-Forsythe testing. Data were compared using Student’s *t*-test (two groups) or by one-way analysis of variance followed by an appropriate multiple comparisons test (more than two groups). Data are expressed as the mean ± SEM. An alpha level of *P* < 0.05 was employed for significance testing.

### Supplementary information

Supplementary legends

Table S1

Figure S1

Figure S2

Figure S3

Figure S4

Figure S5

Figure S6

Uncropped western blots

## Data Availability

The RNAseq datasets generated during the current study have been deposited at National Center for Biotechnology Information (NCBI) Sequence Read Archive (SRA) (https://www.ncbi.nlm.nih.gov/sra) with BioProject accession number PRJNA866091 and the accession number SRR2085014, SRR2085015, SRR2085016, SRR2085017, SRR2085018, SRR2085019.
