# Supplementary material for: The NONRATT023402.2/rno-miR-3065-5p/NGFR axis affects levodopa-induced dyskinesia in a rat model of Parkinson’s disease
Source: Cell Death Discov. 2023 Sep 15;9:342. doi: 10.1038/s41420-023-01644-2 (PMC10504256; doi:10.1038/s41420-023-01644-2)
Supplement: Supplementary file 1 — Supplementary legends [file 41420_2023_1644_MOESM1_ESM.docx]

**Figure Legends**

**Fig. S1** **Immunofluorescence of c-Fos.** A. Immunofluorescence of c-Fos in the right striatum of the Sham group, PD group, and LID group rats. B. Counts of c-Fos positive cells in the right striatum of rats in the Sham, PD, and LID group (n = 3). Data represent the mean ± SEM. ^*^P < 0.05; ^**^P < 0.01.

**Fig. S2 DEGs identified by sequencing and their functional annotation classification.** A. The differential expression of the mRNA volcano plot of the right striatum of Sham + LID and LncRNA + LID group rats (log_2_fold-change > 1.2 and P < 0.05) (n = 3). B. Gene Ontology annotation and classification of DEGs. C. KEGG pathway annotation and classification of DEGs.

**Fig. S3 rno-miR-3065-5p and NGFR levels in the right striatum of LID rats treated with L-DOPA for 0, 2, 4, 6, 8, and 10 weeks (n = 4−5).**

**Fig. S4 Knocking-down of rno-miR-3065-5p aggravated the AIM and activated the PI3K/ Akt pathway of LID rats.** A. Experimental timeline of sham group and microRNA group. B. Relative levels rno-miR-3065-5p in the right striatum of rats injected with the empty vector, and 2 μL AAV- rno-miR-3065-5p-inhibitor. (n = 3) C. Time course of AIM development during the chronic L-DOPA treatment period of Sham group, and microRNA group rats (n = 7). D, E. Time course of the AIM score after a single injection of L-DOPA (treatment on days 9 and 18) of Sham and microRNA group rats (n =7). F, G. Quantification of NGFR, p-PI3K, PI3K, p-Akt, Akt, and c-Fos expressions in the right striatum of Sham, and microRNA group rats (n = 3). The signal intensities of protein bands were normalized to that of GAPDH. Data represent the mean ± SEM. ^*^P < 0.05; ^**^P < 0.01; ^***^P < 0.001.

**Fig. S5 *NGFR* knockdown inhibits the PI3K/Akt signaling pathway and c-Fos in primary neurons.** A. The expression levels of NGFR in the primary neuron cells transfected with NGFR and the siRNA control as determined by RT-PCR are shown. GAPDH was used as a loading control. B, C. Quantification of NGFR, p-PI3K, PI3K, p-Akt, Akt, and c-Fos expressions in the Primary striatal neurons (n = 3). The signal intensities of protein bands were normalized to that of GAPDH. Data represent the mean ± SEM. ^*^P < 0.05; ^**^P < 0.01; ^***^P < 0.001.

**Fig. S6** **Prediction of potential transcription factor c-Fos binding to the promotor of NONRATT023402.2.** A. Potential binding of c-Fos and the promotor of NONRATT023402.2 was predicted by JASPAR. B. The PROMO prediction web tool showed c-Fos regulating NONRATT023402.2.

**Table Legends**

**Table S1. Predicted binding miRNAs and their up-regulated target genes.**
