# Supplementary material for: The NONRATT023402.2/rno-miR-3065-5p/NGFR axis affects levodopa-induced dyskinesia in a rat model of Parkinson’s disease
Source: Cell Death Discov. 2023 Sep 15;9:342. doi: 10.1038/s41420-023-01644-2 (PMC10504256; doi:10.1038/s41420-023-01644-2)
Supplement: Supplementary file 2 — Table S1 [file 41420_2023_1644_MOESM2_ESM.docx]

Table S1. Predicted binding miRNAs and their up-regulated target genes.

| miRNA | Target Score | Target genes | Log2FC | P value |
| --- | --- | --- | --- | --- |
| rno-miR-350 | 88 | ADAMTS17 | 1.443454 | 0.002367 |
|  |  | B3GNT5 | 3.348696 | 9.04E-06 |
|  |  | CARD14 | 1.579482 | 0.007878 |
|  |  | MYO3B | 2.814541 | 1.71E-05 |
|  |  | TMEM30B | 2.008966 | 0.00016 |
|  |  | TRDN | 1.962769 | 0.004872 |
|  |  | UNC45B | 1.509221 | 0.000417 |
|  |  | VDR | 4.674199 | 7E-13 |
| rno-miR-29a-5p | 73 | CHST4 | 3.119426 | 3.67E-05 |
|  |  | IQCH | 2.561994 | 0.000554 |
|  |  | NXPH4 | 1.366269 | 9.35E-06 |
|  |  | PCOLCE2 | 1.724864 | 0.006877 |
|  |  | PDE6B | 1.872502 | 0.002492 |
|  |  | POU1F1 | 2.612625 | 0.015316 |
|  |  | TNFRSF9 | 1.214767 | 0.028419 |
| rno-miR-509-3p | 72 | ADAMTS16 | 2.84502 | 0.002187 |
|  |  | HPD | 1.363883 | 0.043034 |
|  |  | MOGAT2 | 1.407215 | 0.002713 |
|  |  | RBM20 | 1.447869 | 0.006605 |
| rno-miR-551b-5p | 67 | IKZF3 | 1.65841 | 0.005069 |
|  |  | LMOD3 | 2.140552 | 0.000406 |
|  |  | PRG4 | 3.060821 | 0.001487 |
|  |  | SPINK8 | 2.136705 | 0.047827 |
|  |  | TPBG | 2.145111 | 2.77E-16 |
| rno-miR-20a-3p | 63 | NPNT | 1.499779 | 0.001117 |
|  |  | PDYN | 2.072953 | 2.42E-13 |
| rno-miR-3065-5p | 63 | PTGFR | 1.433586 | 0.046127 |
|  |  | NPNT | 1.499779 | 0.001117 |
|  |  | NGFR | 1.289657 | 0.021041 |
| rno-miR-7a-1-3p | 57 | NMU | 2.334778 | 0.001704 |
|  |  | ELK4 | 2.100866 | 0.000157 |
|  |  | ACTRT3 | 3.828849 | 0.004201 |
|  |  | GYS2 | 2.315773 | 0.007392 |
|  |  | TSHZ3 | 1.333577 | 0.000136 |
|  |  | SLC5A12 | 1.67088 | 0.02038 |
|  |  | L3MBTL4 | 2.455752 | 0.005201 |
| rno-let-7c-1-3p | 54 | BANK1 | 2.229468 | 0.004713 |
|  |  | C2CD4A | 2.498733 | 0.013428 |
|  |  | C2CD4B | 2.433518 | 0.033724 |
|  |  | GRK1 | 1.832994 | 0.048571 |
|  |  | INHBA | 1.387747 | 4.52E-05 |
|  |  | PCOLCE2 | 1.724864 | 0.006877 |
|  |  | PLEK2 | 2.262432 | 6.99E-05 |
|  |  | ROBO3 | 2.060371 | 0.006517 |
|  |  | SCG2 | 1.3697 | 2.69E-07 |
|  |  | SEMA3C | 1.328041 | 1.52E-06 |
|  |  | SSTR2 | 1.380183 | 0.001179 |
|  |  | VDR | 4.674199 | 7E-13 |
| rno-miR-463-3p | 53 | DIAPH3 | 1.88409 | 0.004814 |
|  |  | KLHL1 | 1.353426 | 0.042349 |
|  |  | SRM | 1.70625209 | 0.0000226 |
