# Supplementary figures and images for: The NONRATT023402.2/rno-miR-3065-5p/NGFR axis affects levodopa-induced dyskinesia in a rat model of Parkinson’s disease
Source: Cell Death Discov. 2023 Sep 15;9:342. doi: 10.1038/s41420-023-01644-2 (PMC10504256; doi:10.1038/s41420-023-01644-2)

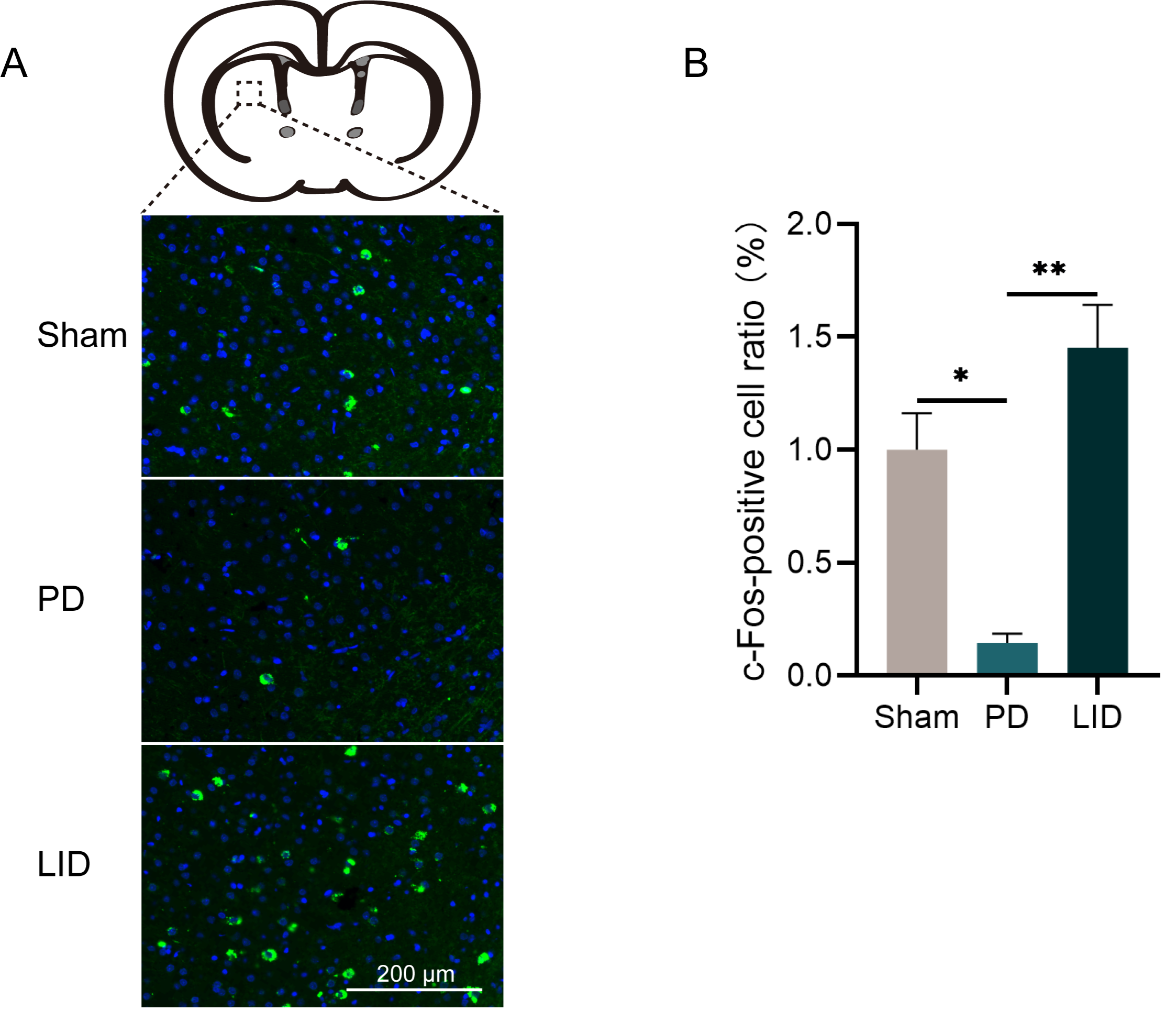

Supplement: Supplementary file 3 — Figure S1 [file 41420_2023_1644_MOESM3_ESM.tif]

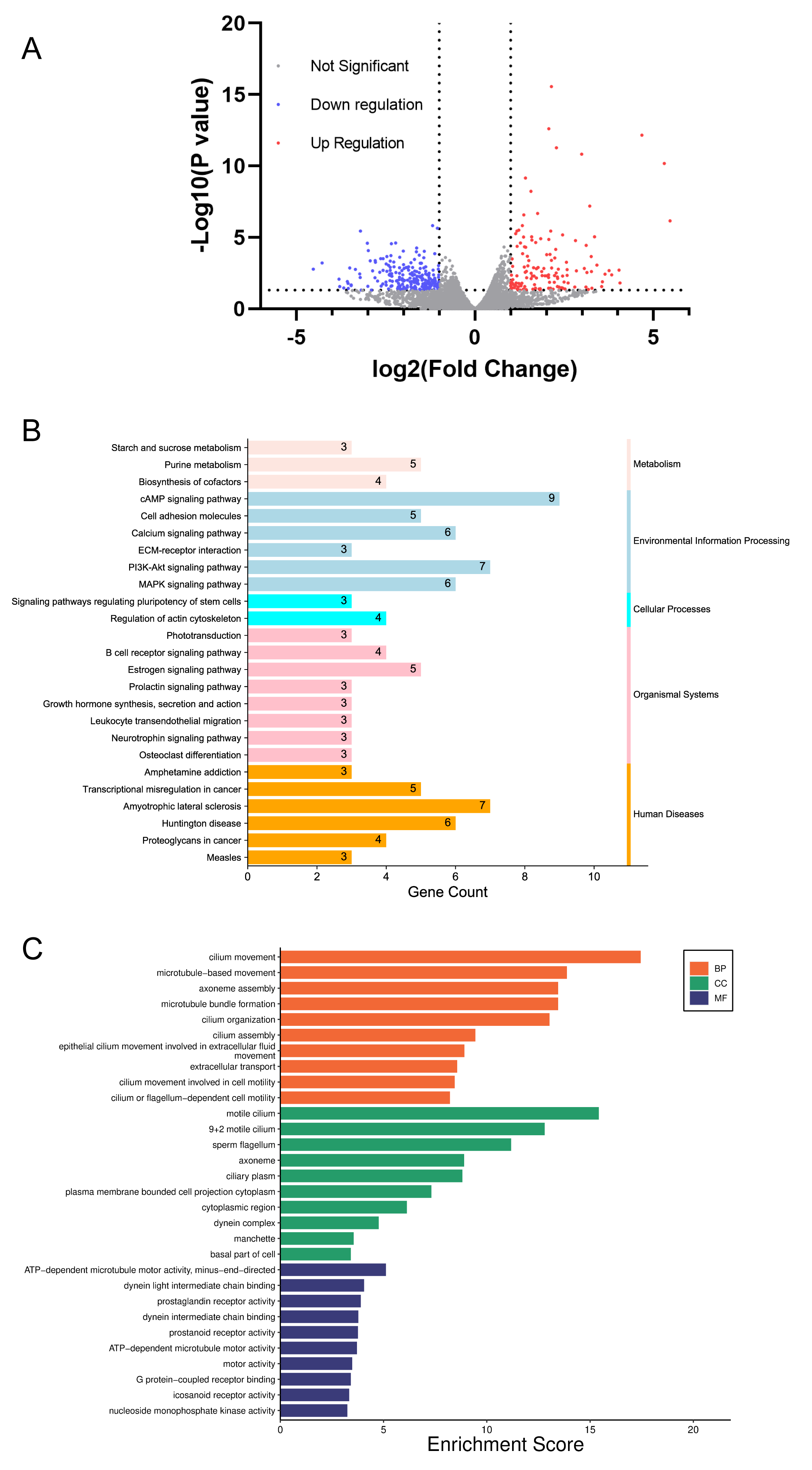

Supplement: Supplementary file 4 — Figure S2 [file 41420_2023_1644_MOESM4_ESM.tif]

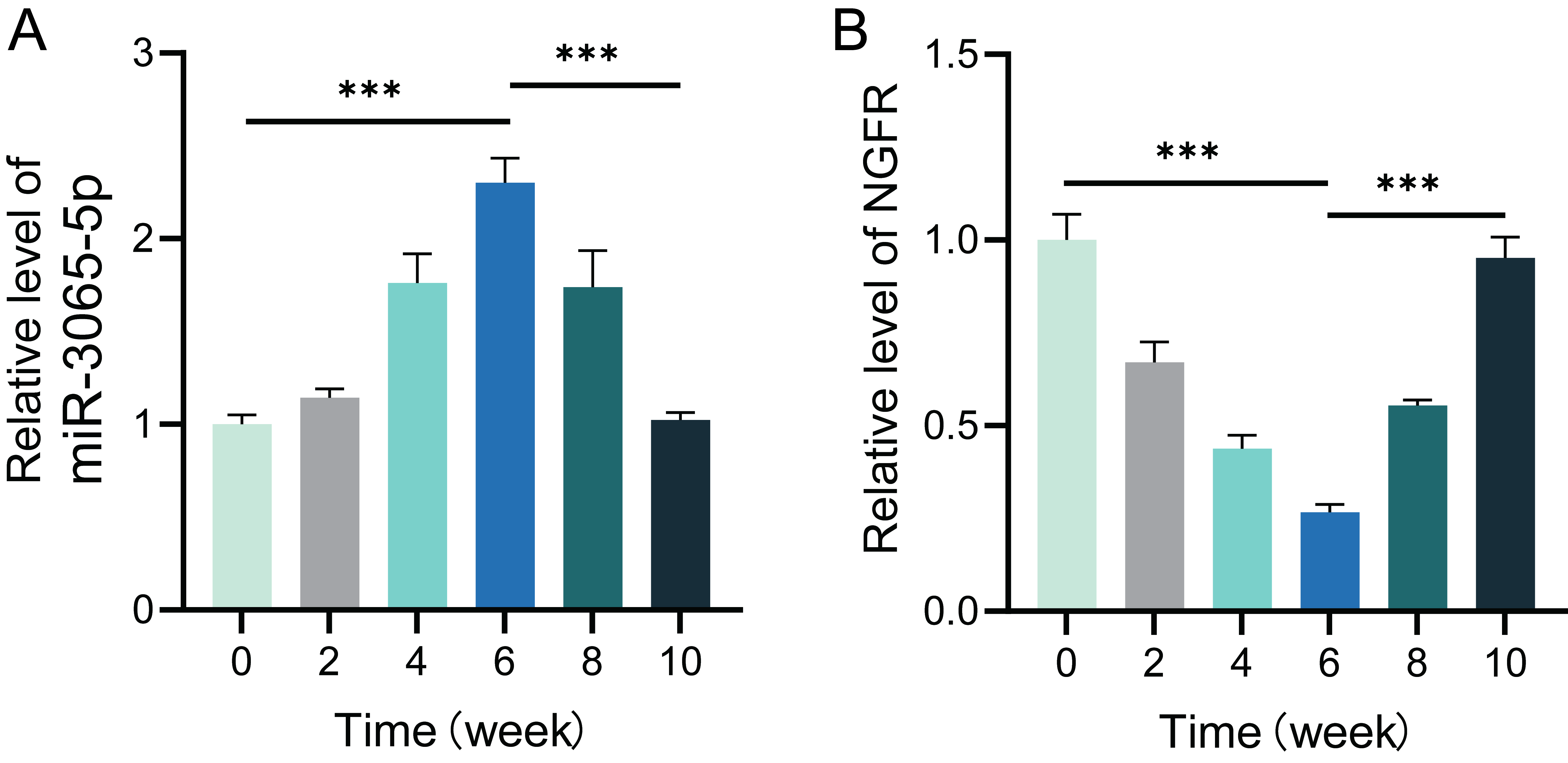

Supplement: Supplementary file 5 — Figure S3 [file 41420_2023_1644_MOESM5_ESM.tif]

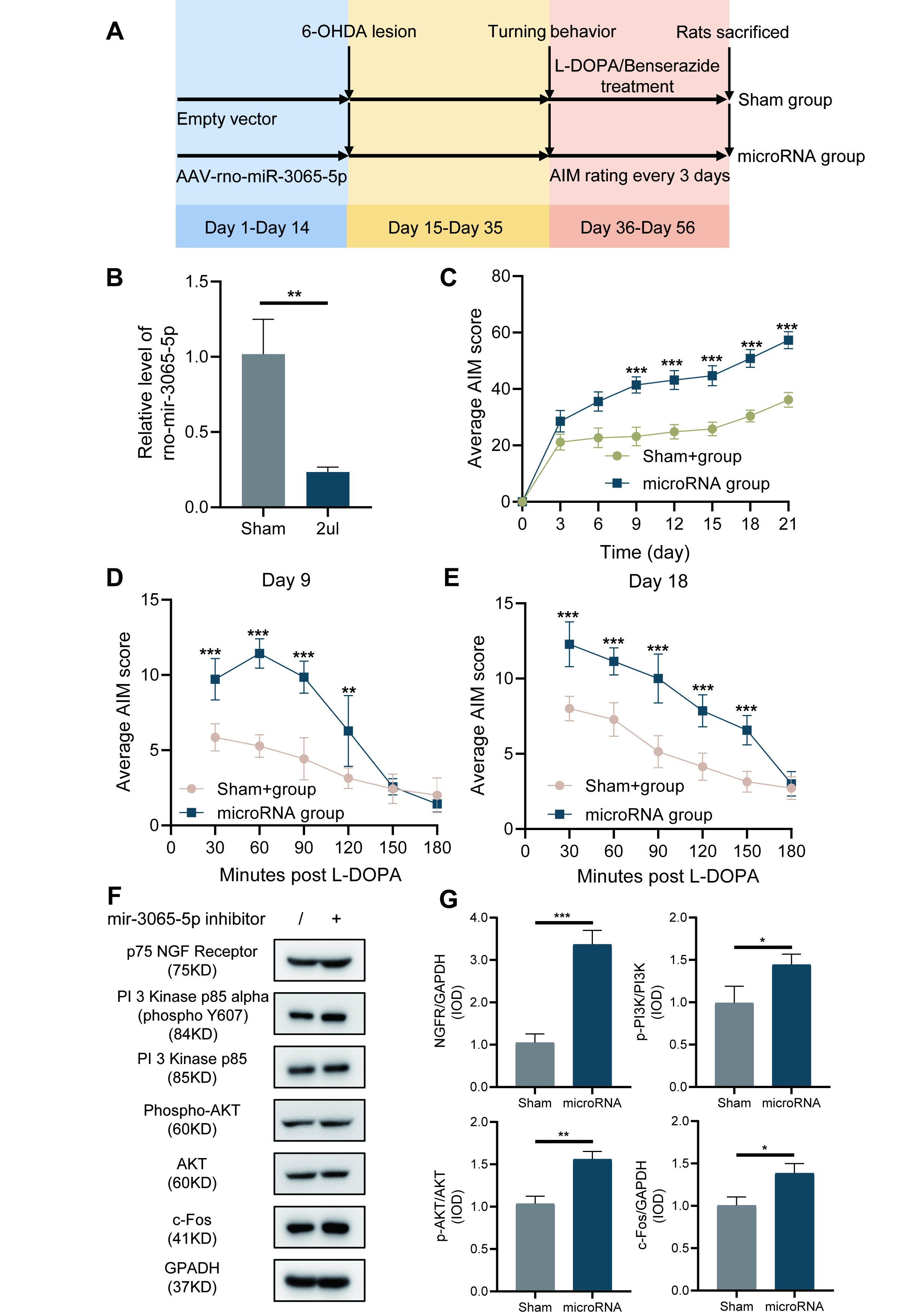

Supplement: Supplementary file 6 — Figure S4 [file 41420_2023_1644_MOESM6_ESM.tif]

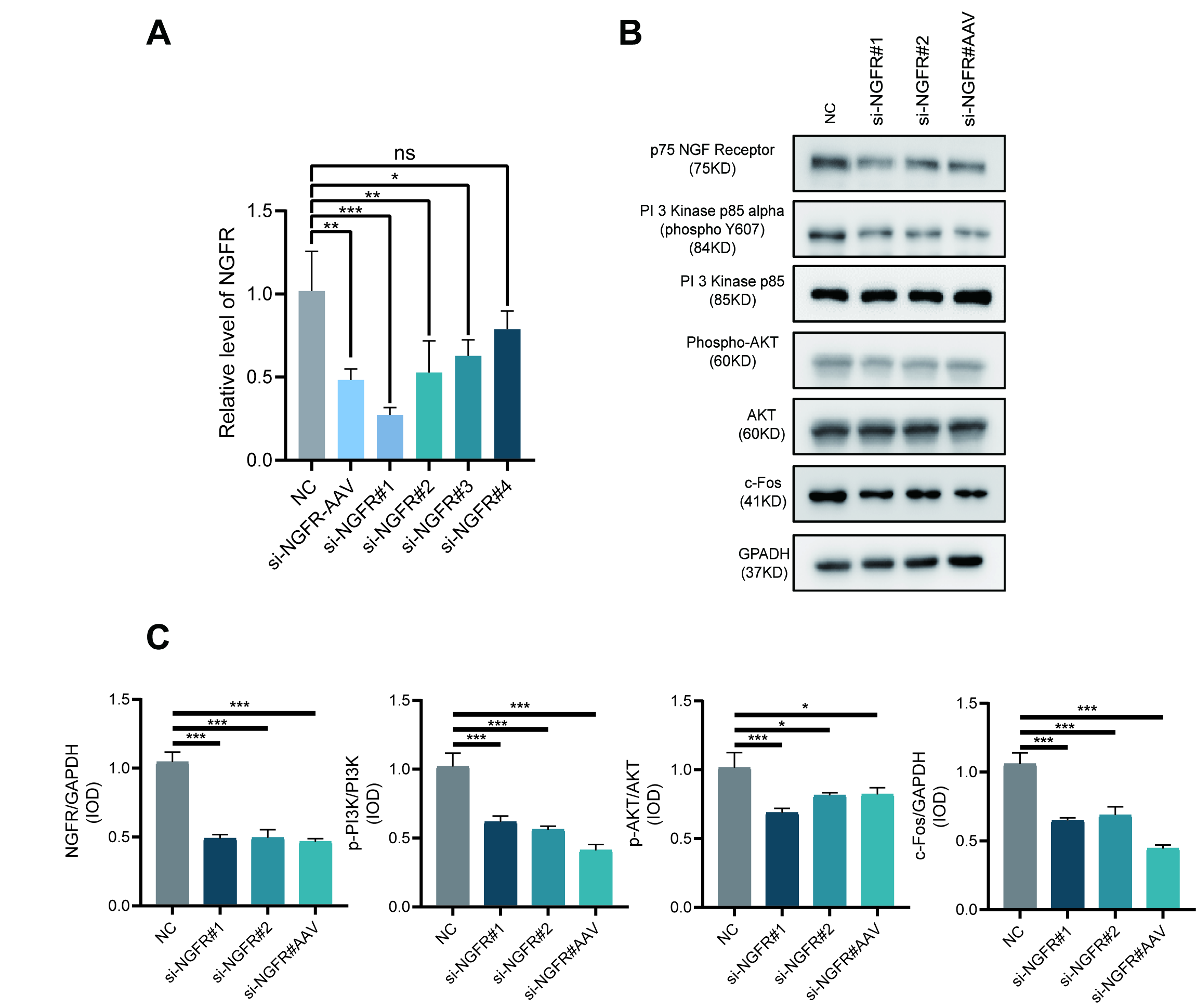

Supplement: Supplementary file 7 — Figure S5 [file 41420_2023_1644_MOESM7_ESM.tif]

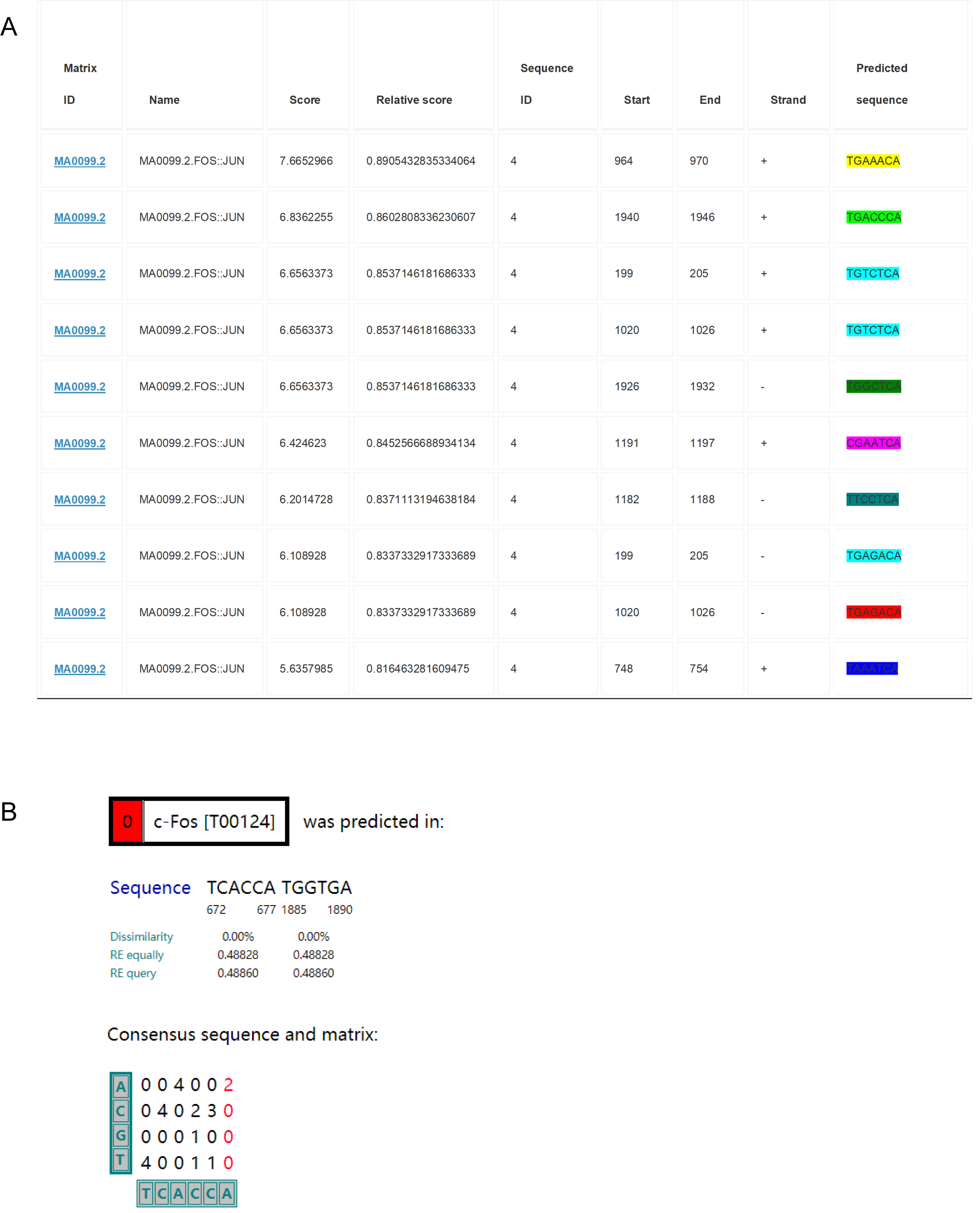

Supplement: Supplementary file 8 — Figure S6 [file 41420_2023_1644_MOESM8_ESM.tif]

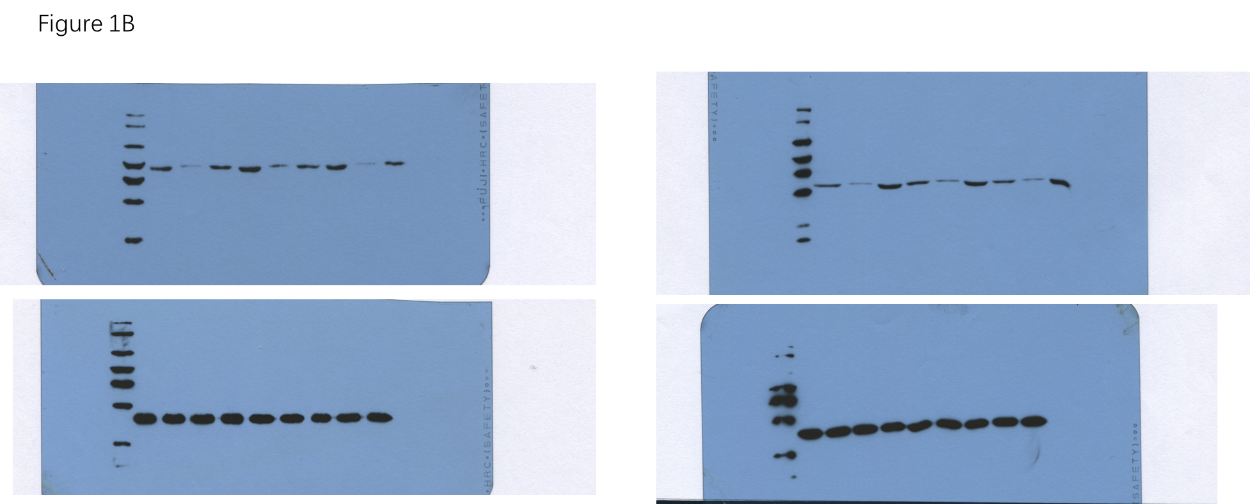


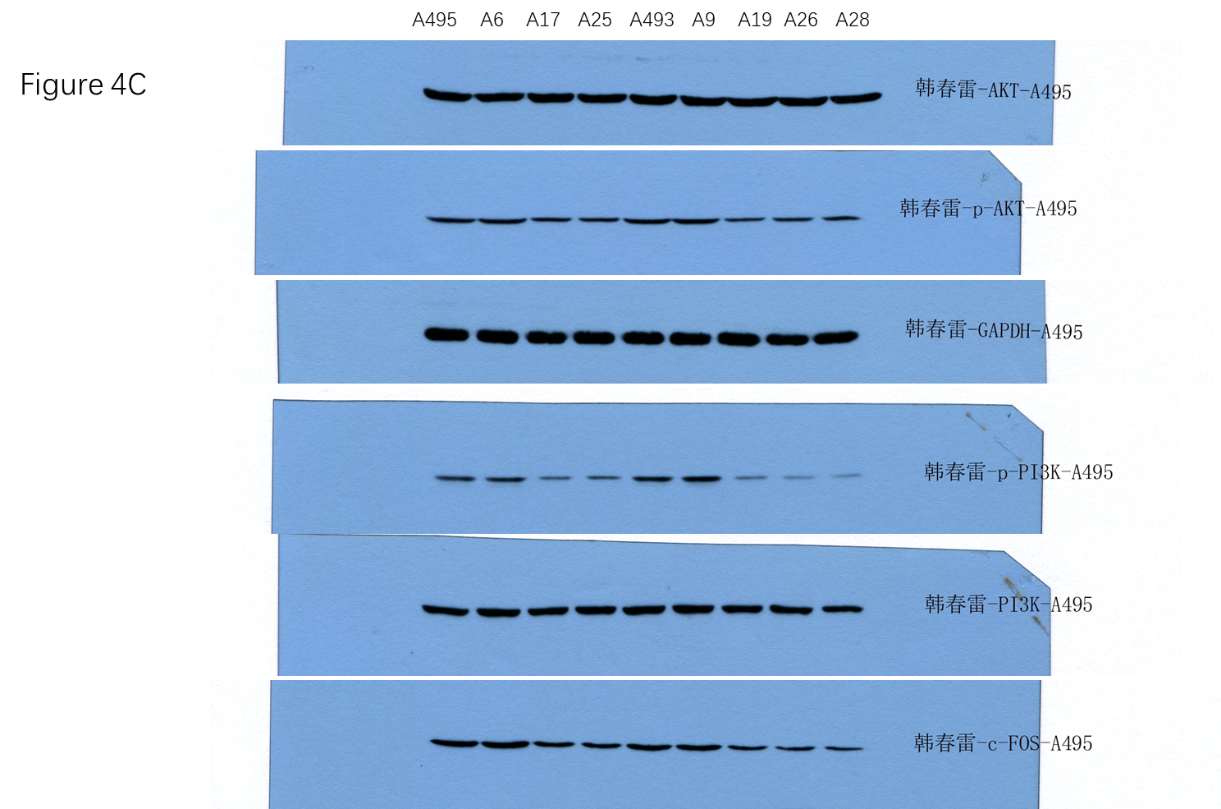

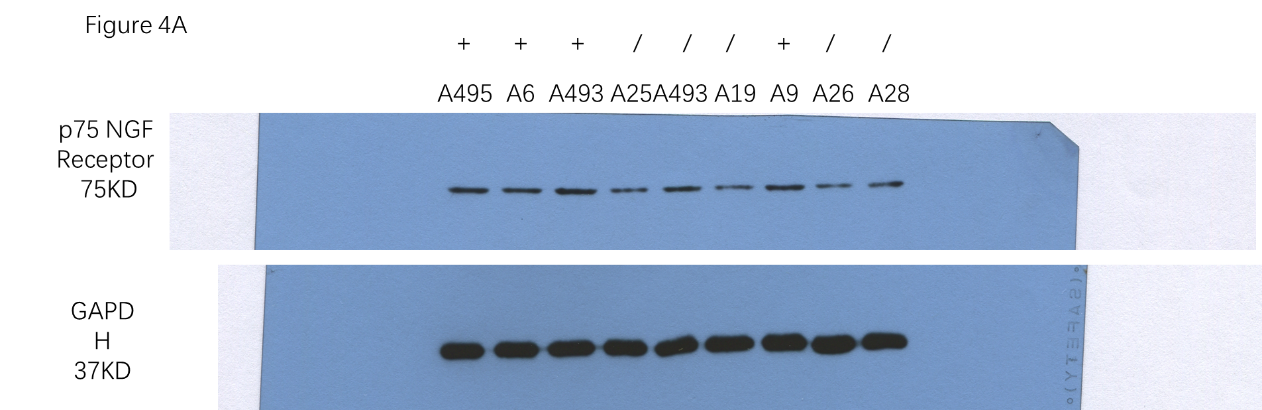


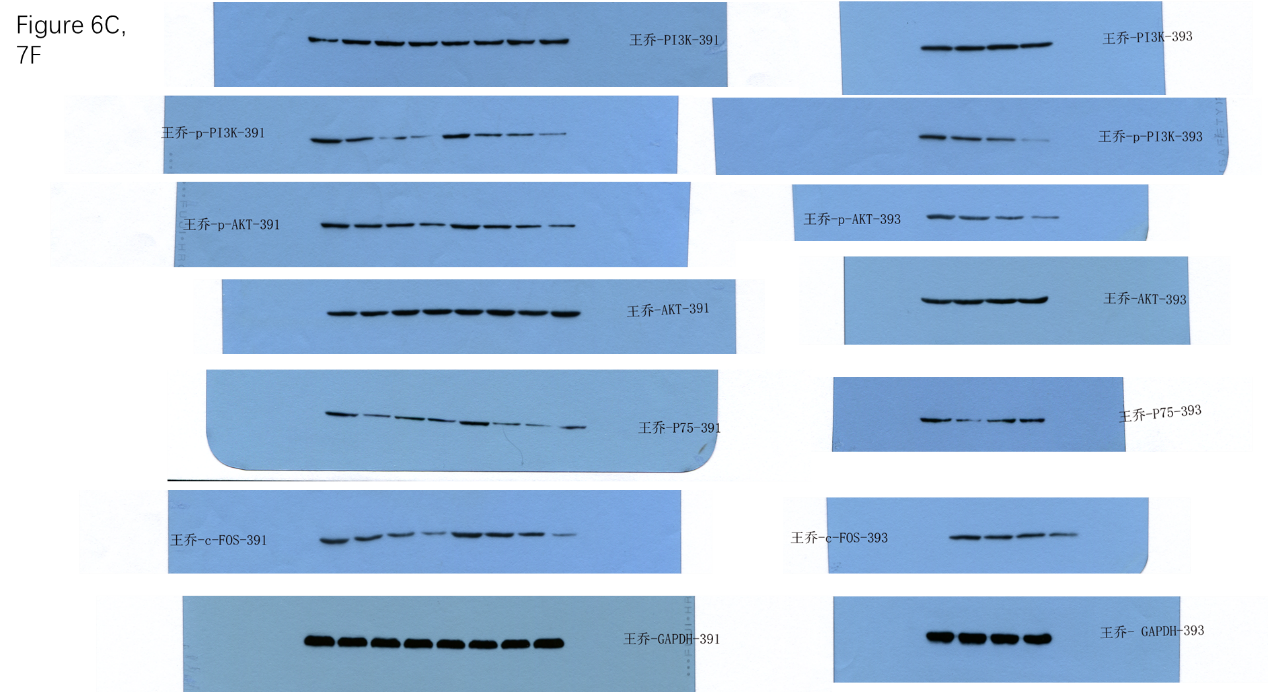


**Fig. S4F**


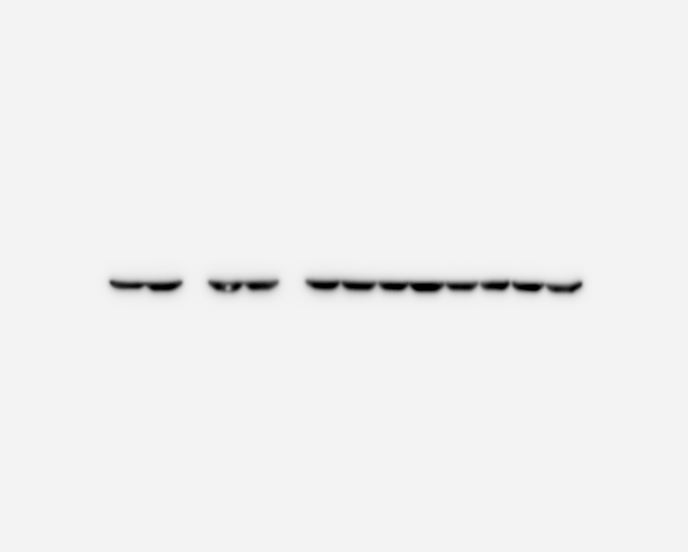


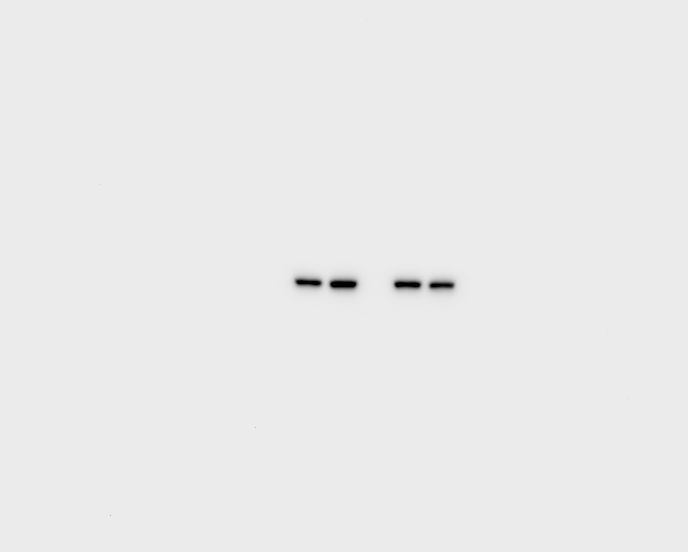


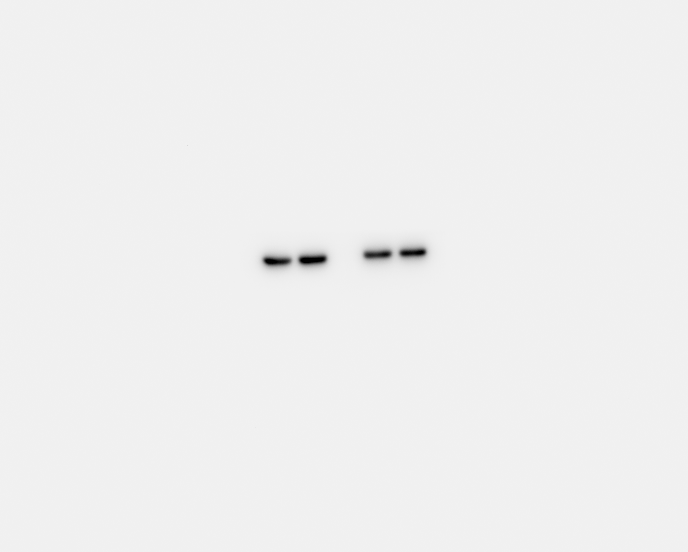


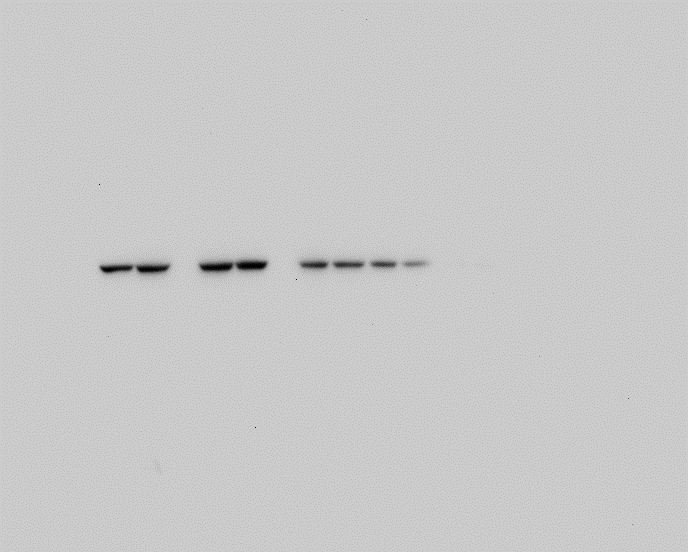

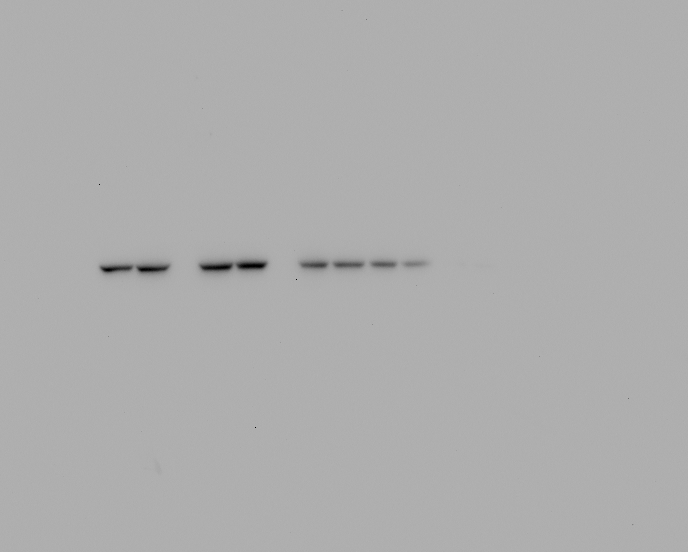


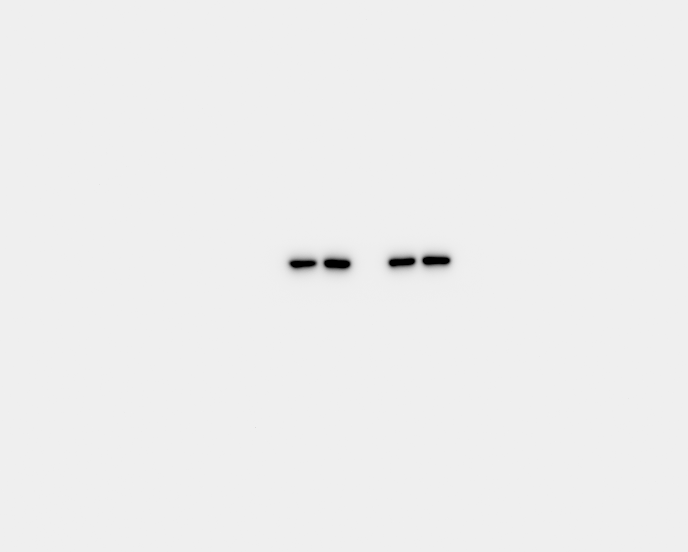


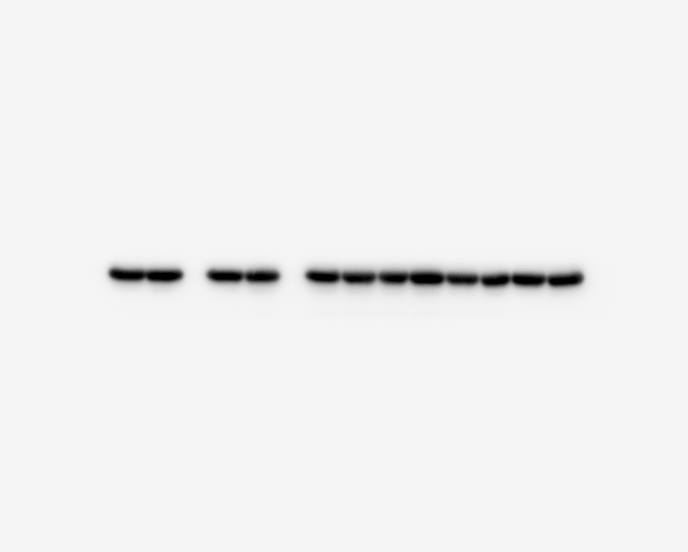


**Fig S5B**


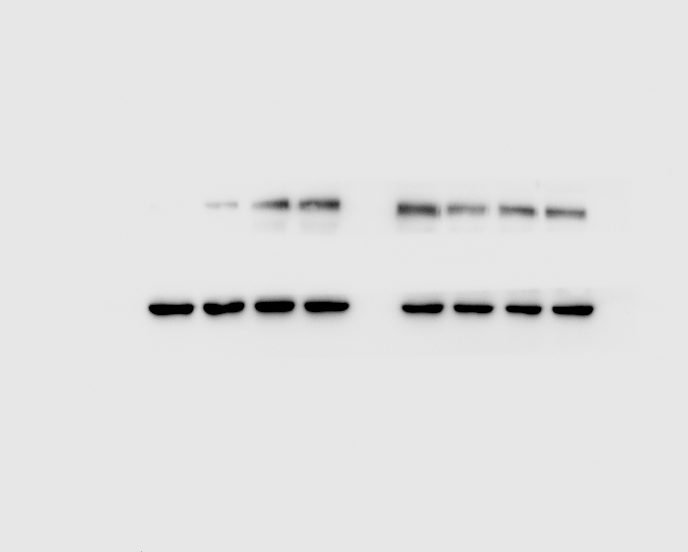


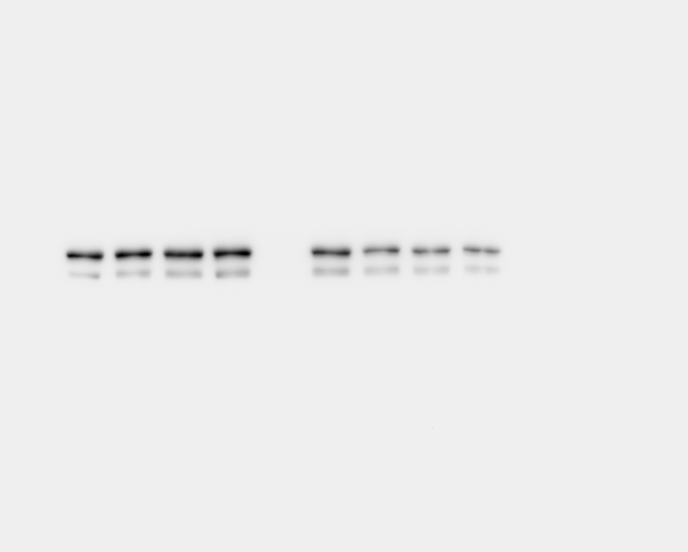


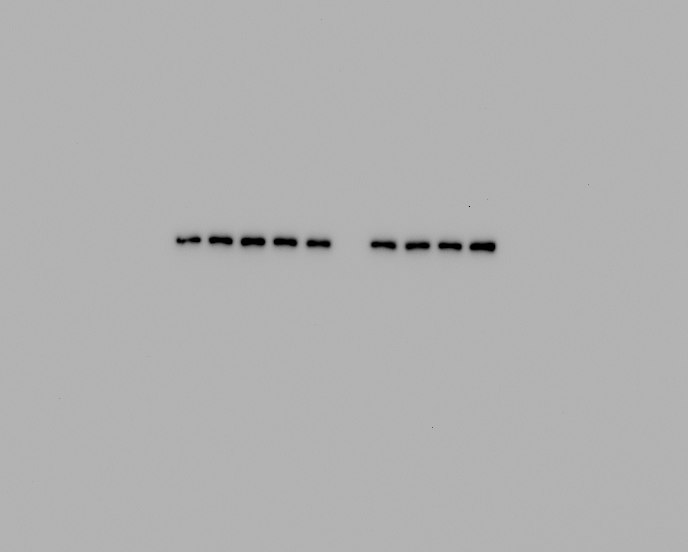


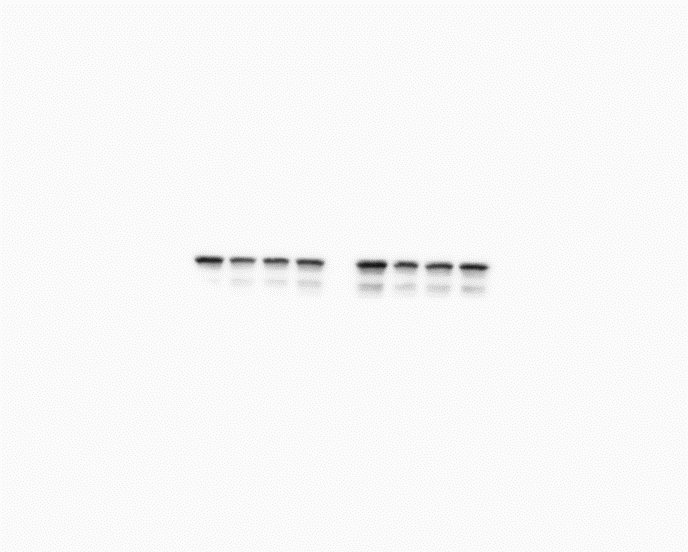


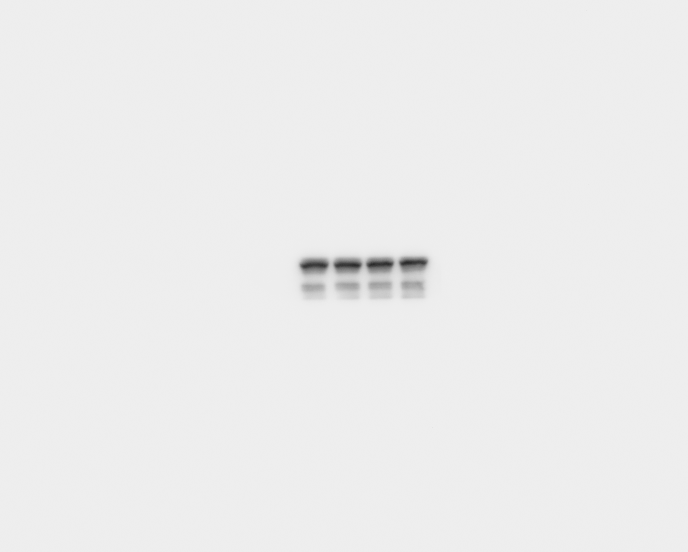


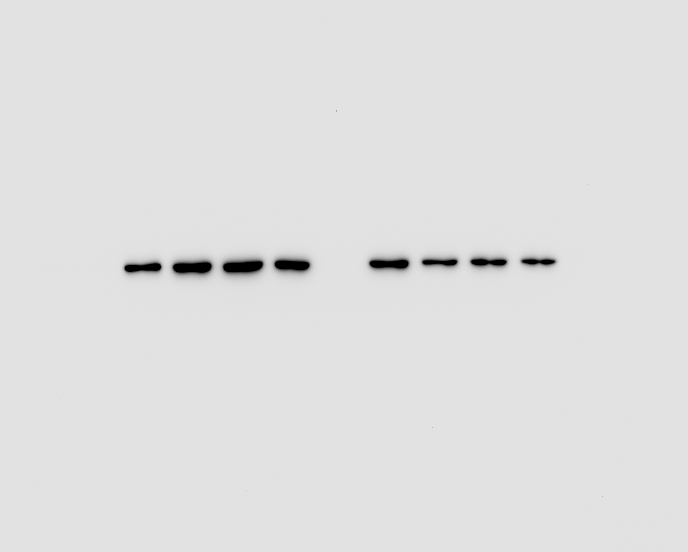


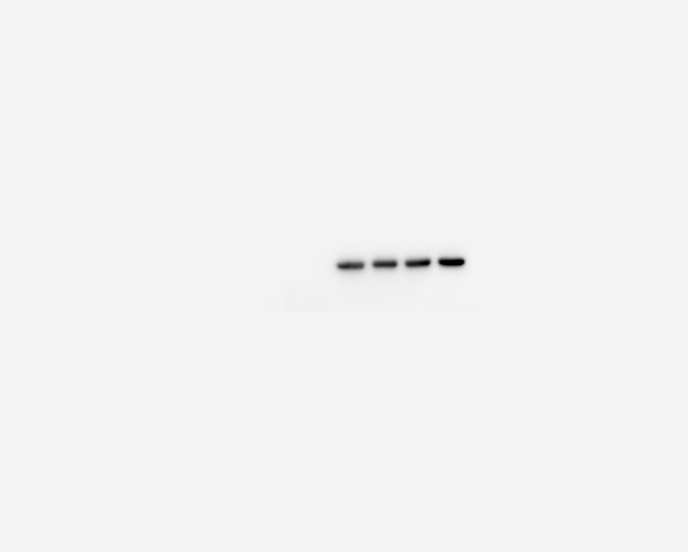

Supplement: Supplementary file 9 — Uncropped western blots [file 41420_2023_1644_MOESM9_ESM.docx]
